# High-Integrity Sequencing of Spike Gene for SARS-CoV-2 Variant Determination

**DOI:** 10.3390/ijms23063257

**Published:** 2022-03-17

**Authors:** Yu-Chieh Liao, Feng-Jui Chen, Min-Chieh Chuang, Han-Chieh Wu, Wan-Chen Ji, Guann-Yi Yu, Tsi-Shu Huang

**Affiliations:** 1 Institute of Population Health Sciences, National Health Research Institutes, No. 35, Keyan Road, Zhunan Town, Miaoli County 35053, Taiwan; 2 National Institute of Infectious Diseases and Vaccinology, National Health Research Institutes, No. 35, Keyan Road, Zhunan Town, Miaoli County 35053, Taiwan; frchen@nhri.edu.tw (F.-J.C.); hanjie@nhri.edu.tw (H.-C.W.); guannyiy@nhri.edu.tw (G.-Y.Y.); 3 Department of Biological Science and Technology, National Yang Ming Chiao Tung University, Hsinchu 30010, Taiwan; 4 Department of Chemistry, Tunghai University, Taichung 40704, Taiwan; mcchuang@thu.edu.tw (M.-C.C.); ss870805ss@gmail.com (W.-C.J.); 5 Division of Microbiology, Department of Pathology and Laboratory Medicine, Kaohsiung Veterans General Hospital, Kaohsiung 81362, Taiwan; tshuang@vghks.gov.tw

**Keywords:** SARS-CoV-2, variant, spike gene, nanopore sequencing

## Abstract

For tiling of the SARS-CoV-2 genome, the ARTIC Network provided a V4 protocol using 99 pairs of primers for amplicon production and is currently the widely used amplicon-based approach. However, this technique has regions of low sequence coverage and is labour-, time-, and cost-intensive. Moreover, it requires 14 pairs of primers in two separate PCRs to obtain spike gene sequences. To overcome these disadvantages, we proposed a single PCR to efficiently detect spike gene mutations. We proposed a bioinformatic protocol that can process FASTQ reads into spike gene consensus sequences to accurately call spike protein variants from sequenced samples or to fairly express the cases of missing amplicons. We evaluated the in silico detection rate of primer sets that yield amplicon sizes of 400, 1200, and 2500 bp for spike gene sequencing of SARS-CoV-2 to be 59.49, 76.19, and 92.20%, respectively. The in silico detection rate of our proposed single PCR primers was 97.07%. We demonstrated the robustness of our analytical protocol against 3000 Oxford Nanopore sequencing runs of distinct datasets, thus ensuring high-integrity sequencing of spike genes for variant SARS-CoV-2 determination. Our protocol works well with the data yielded from versatile primer designs, making it easy to determine spike protein variants.

## 1. Introduction

The first cases of SARS-CoV-2 emerged in late 2019 and, thus far, it has caused over 450 million cases and 6 million deaths worldwide [1]. Cases and hospitalizations have continued to increase, and to date, more than 63% of the world population has received at least one dose of a COVID-19 vaccine [2,3]. The inherent property of viruses to achieve immune escape has induced numerous mutations in the spike protein of SARS-CoV-2, which is responsible for virus–cell binding and is the target for virus-neutralising antibodies [4,5,6,7]. Many vaccines are designed to generate antibodies against the spike protein [4,5,6,7]. Recently, five SARS-CoV-2 lineages were selected as variants of concern by the World Health Organization; these lineages have the following pronounced mutations in the spike protein: HVdel69-70, Ydel144, N501Y, A570D, D614G, P681H, T716I, S982A, and D1118H [8] of B.1.1.7 (Alpha); L18F, D80A, D215G, LALdel242-244, R246I, K417N, E484K, N501Y, D614G, and A701V [5,9] of B.1.351 (Beta); L18F, T20N, P26S, D138Y, R190S, K417N, E484K, N501Y, D614G, H655Y, and T1027I [10] of P.1 (Gamma); and T19R, K77R, G142D, EFdel156-157, R158G, A222V, L452R, T478K, D614G, P681R, and D950N [11] of B.1.617.2 (Delta); the Omicron variant is exceptional for carrying over 30 mutations in the spike glycoprotein [12]. Thus, for the intensive surveillance of SARS-CoV-2, the spike gene must be continuously monitored [13]. Accordingly, sequencing the whole viral genome (~30 kbp) of SARS-CoV-2 remains the most practical method to determine the sites of mutation. The first SARS-CoV-2 genome, isolated in Wuhan, China, and published in January 2020 [14], has facilitated the design of tiling PCR approaches for whole-genome sequencing [15,16,17] or molecular detection [18]. The ARTIC Network provided a V3 protocol using 98 primer pairs for amplicon production [19], and it was the widely used amplicon-based approach for rapid tiling of the SARS-CoV-2 genome. However, ARTIC amplification has regions of low or missing sequence coverage [20,21]. With the emerging Beta and Delta variants, some of the ARTIC V3 primers have stopped working; therefore, ARTIC V4 primers were designed in June 2021 [22]. Moreover, approximately 19–43% of the SARS-CoV-2 genomes generated monthly were gapped, and unprecedented genome sequencing efforts are ongoing worldwide [23]. It therefore remains unclear whether such an amplicon tiling approach is appropriate for sequencing spike genes. Although whole-genome sequencing is a popular method to fully understand viral transmission and evolution, this ARTIC method of using 99 (V4) primer pairs and two separate multiplex PCRs is labour-, time-, and cost-intensive. Consequently, it may be inadequate to meet the demands of high-load sequencing. A less complicated approach for sequencing the spike gene is warranted to acquire essential mutation information on viral variants.

The Oxford Nanopore sequencing technique, which is effective for long-sequence sequencing, may be a promising approach to more easily sequence the spike gene. This technique requires substantially fewer primers [24,25]. In this study, we evaluated the in silico detection rate of primer sets that yield amplicon sizes of 400 [19,22], 1200 [21], and 2500 bp [15] for genome and spike gene sequencing of SARS-CoV-2. In contrast to the previous method [15], which requires two primer pairs in two separate PCRs to obtain spike gene sequences from the 2500-bp amplicons, we proposed a single PCR requiring dual one-base-overlapping amplicons for efficient detection of spike gene mutations. To detect the variants and low-coverage sequencing regions [26], we proposed a bioinformatic protocol that can process FASTQ reads into spike gene consensus sequences to accurately call spike protein variants from sequenced samples or to adequately express the cases of missing amplicons. We demonstrated the robustness of this protocol against distinct datasets (3000 sequencing runs from Sequence Read Archive (SRA)), ensuring high-integrity sequencing of spike genes for variant SARS-CoV-2 determination.

## 2. Results

### 2.1. Disadvantages of Small-Amplicon Tiling Sequencing

**Figure 1:**
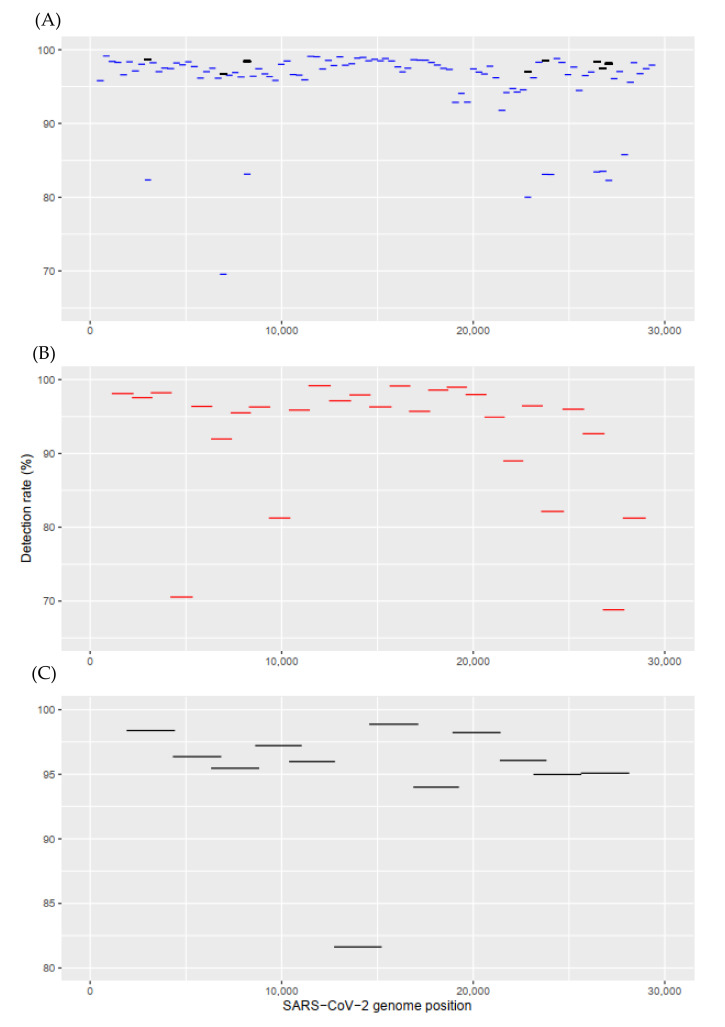
In silico evaluation of primer sets across the SARS-CoV-2 genome. (**A**) Set I: ARTIC nCoV-2019 V4, updated primers (primer pairs 10, 23, 27, 76, 79, 88, 89, and 90 were updated in V4.1) in black lines, (**B**) set II: SARS-CoV-2-Midnight, and (**C**) set III: 1.4 × 2.5 kb amplicons. An amplicon-wise detection rate corresponding to each primer pair was determined: No. of genome containing the sequences of primer pair/Total number of genomes 848,003 SARS-CoV-2 genome sequences downloaded from NCBI on 14 February 2022.

To synthesise the SARS-CoV-2 genome-equivalent amplicons for tiling sequencing, three primer sets were explored, which contain 99, 29, and 14 primer pairs in connection to the resulting amplicon length of 400 bp [9,16,19,22,27,28,29,30], 1200 bp [21,31], and 2500 bp [15,32], respectively (Table 1). We examined in silico these primer sets against 848,003 complete SARS-CoV-2 sequences in the NCBI database (14 February 2022) and considered that PCR products were only obtained when both the paired primer sequences were matched to the genome of the target. The number of targeted genomes containing specific primer pairs was counted. An amplicon-wise detection rate corresponding to each primer pair was determined (Figure 1). For primer set I, the detection rates of V4 primers ranged from 69.56% to 99.14%; however, only 81,279 (9.58%) of the targeted genomes contained all 99 primer pairs. Some primers were updated in V4.1 (black lines in Figure 1A), but the targeted genomes were only increased to 123,195 (14.53%). This suggests that primer set I is unlikely to obtain high-quality consensus genomes (without Ns). Although primer sets II and III yielded higher values, their detection rates were unsatisfactory at 28.30% and 58.53%, respectively (Table 1). Particularly for the spike gene, 14, 4, and 2 primer pairs were required for the three primer sets to yield the corresponding rates at 59.49%, 76.19%, and 92.20% (Table 1). Primer set I was incapable of offering complete information about the spike gene.

**Figure 2:**
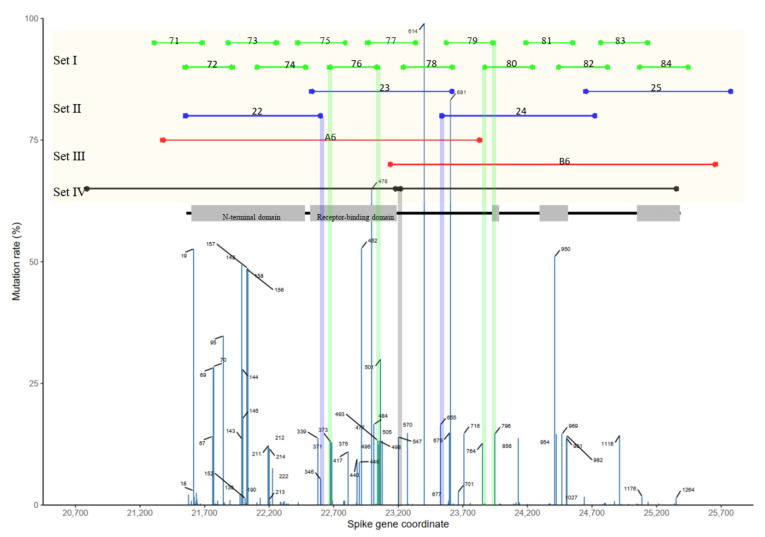
Coordinates of primer sets and the mutation rate of spike protein based on the hCov19 Mutation Dashboard on 12 February 2022. Green, blue, red, and black horizontal lines represent primer set I, set II, set III, and our primer set (set IV) covering the spike gene of SARS-CoV-2, respectively.

We examined the mutation rate of the spike protein based on the hCov19 Mutation Dashboard (GISAID) on 12 February 2022 [33] (Figure 2). Of the 7,919,209 sequences, mutation occurrences of more than 1000 were retrieved to exploit 446 variant sites of amino acids. The mutation rate corresponding to a certain amino acid was calculated by dividing its occurrence by 7,919,209. Figure 2 presents the sites with a mutation rate of >0.2% and the position-labelled sites with a mutation rate of >1%. Aligning the primer number per spike gene coordination revealed overlaps (in genetic sequence) of primers: 76F with mutation site 371 (12.79%), 76R with triple variant sites 493 (13.31%), 496 (12.36%) and 498 (13.14%), 79R with variant site 796 (14.61%), and primer 80F with variant site 764 (12.72%), leading to inefficient amplicon production and poor-quality tiling sequencing using primer set I (V4). Recently, ARTIC updated primers including 76F, 76R and 79R in V4.1 for the Omicron variant in December 2021, but the inefficient primers 71F and 80F (as evident in Figure 1A) could not produce amplicons for covering the complete spike gene; the percentage of the genome containing spike gene primers marginally increased from 57.87% (primer pairs: 71–84 in V4) to 59.49% (V4.1), as shown in Table 1. Amplification failure is likely to preclude the recognition of highly probable mutation sites such as 452, 477, and 484 [34]. Similarly, the variant sites of 346 (5.30%) and 655 (16.42%) could account for the primer inefficiency of 22R and 24F in primer set II. As the mutations increase in the viral genome, the efficiency and quality of the multiple-primer set deteriorate.

### 2.2. Single-Tube PCR for Identifying Spike Protein Mutations

Although primer set III used two primer pairs (A6 and B6, depicted in Figure 2) to cover the SARS-CoV-2 spike gene in connection with a 92.20% detection rate (Table 1), two separate PCRs were required per sample. We designed a single PCR incorporating two primer pairs (S1 and S2) to cover the spike gene (from 20,794 to 25,347); our protocol yielded an in silico detection rate of 97.07%. The two amplicons overlapped in one base (primer set IV, black lines in Figure 2), enabling the realisation of a single reaction without undesired amplicon production brought by crosstalk (e.g., A6R and B6F). Furthermore, the overlapped primers were designed to locate sequences beyond the receptor-binding domain (RBD, from 319 to 541 amino acids), because mutations of key residues in RBD play a vital role in enhancing the interaction with cell receptor ACE2 [35]. The resulting amplicons cover the whole spike gene, except for the 546–554 and 1262–1273 fragments. As indicated in Figure 2, the coordination of primer S2F overlaps the variant site of 547 (13.99%), and two primer sequences were therefore designed for S2F. Accordingly, please note that primer set IV could not detect T547K in Omicron variants. Overall, primer set IV offers the following advantages: (i) enhanced detection rate to 97.07%, (ii) simplicity owing to a single PCR per sample, and (iii) decreased possibility of missing amplicons by using only two primer pairs. Diluted viral RNA solutions and six clinical samples were subsequently used to interrogate the single-tube PCR system (containing primer set IV), and the resulting amplicons were sequenced using Oxford Nanopore MinION.

### 2.3. A bioinformatic Protocol for Spike Consensus Sequence

To enable high-integrity sequencing of the spike gene, we further developed a bioinformatic protocol to assemble the reads generated from the single-reaction amplicons. A bioinformatic protocol that is superior in assembling reads to a complete consensus sequence is of significance for the surveillance of genetic changes in the global virus population. Using a diluted viral RNA solution (corresponding to Ct = 28.07 obtained from a RT-PCR assay, see Supplementary Materials for details) as a target of demonstration revealed that a nearly complete spike protein sequence (1–1261 of 1273 amino acids) was obtained, and that a 9-amino-acid deletion was identified (I68-; H69-; V70-; S71-; G72-; T73-; N74-; G75-; and T76-). A further diluted viral RNA solution (corresponding to Ct = 31.79) was also tested to determine the limit of detection of the system. The generated sequencing alignment yielded a ‘multiple-fragment consensus’ conclusion as a result of discontinuous consensus sequences; the BLAST result referred these consensus segments to SARS-CoV-2 (Supplementary Figure S1). The results revealed that our protocols could explicitly indicate the amino acid variants of the spike protein with a sensitivity superior to or comparable to that yielded by primer set III (Ct = 29.2 [15]). Our proposed primer set IV was used in six clinical samples for single-tube PCR, and the PCR products were then sequenced with MinION. Five Alpha and one Delta variants were successfully identified by our bioinformatic protocol. To verify the finding, the protocol was also interrogated with the 157 Nanopore sequencing data of PRJNA675364 [15]. The results revealed a file named Result.csv, which summarised sample name, variant, nucleotide, and amino acid sequences. As depicted in Supplementary Table S1, 91 samples were identified as having no amino acid mutation in the spike protein, and 58 samples had a single D614G mutation, the substitution that was dominant in the late 2020s and enhances viral replication and transmission [36,37]. Additionally, our analysis protocol indicated that the other eight samples had one or two substitutions. The information provided by our protocol was easily readable, which is superior to the prior method [26]. These characteristics are advantageous for monitoring viral evolution, which contain specified mutation sites and may be harmful to human health.

To underline the mutation-caused amplicon-missing, we used the 64 Nanopore sequencing data of PRJNA694014 [9] as an example of demonstration to be analysed using our bioinformatic protocol. The results clearly indicated the 242–244 deletion site (LALdel242–244) and the five amino acid substitutions (K417N, E484K, N501Y, D614G, and A701V) in 28 samples (Supplementary Table S1), consistent with the conclusion reported in [9]. For the other 36 samples, our results displayed ‘segment-missing amplicon’ or ‘multiple-fragment consensus’ to present the fragments with low amplification efficiency. Notably, the low amplification efficiency led to low sequencing depth (Supplementary Figure S2), which is usually ignored by the ARTIC protocol, thereby providing inaccurate information (e.g., L242H, A243-, L244-, and H245- in Supplementary Table S1). Thus, our protocol substantially avoided the false discovery of sequence mutation.

**Figure 3:**
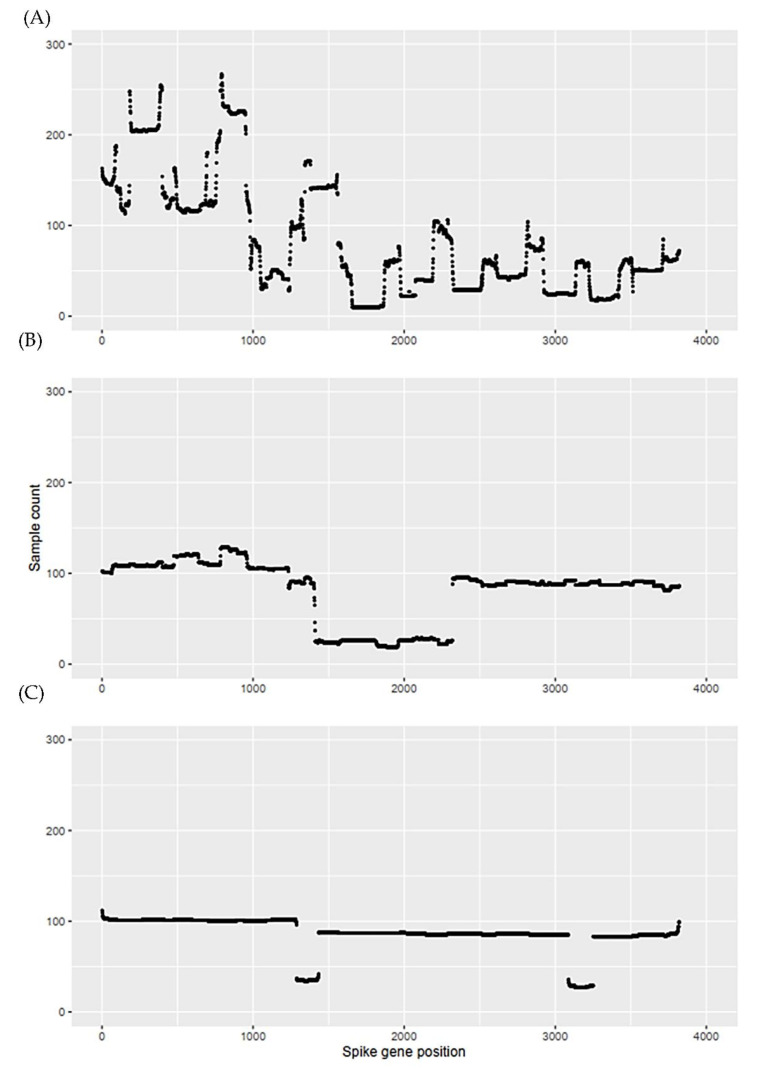
Sample count profiles with sequencing depth <25 across the spike gene. A total of 3000 Nanopore sequencing runs of SARS-CoV-2 samples downloaded from Sequence Read Archive (SRA). One thousand sequencing runs of (**A**) set I with AvgSpotLength < 600 bp; (**B**) set II, 600–1600 bp; and (**C**) set III, ≥1600 bp.

A larger scale of Nanopore sequencing data was also applied to our protocol to understand its performance and the disadvantages of small-amplicon tiling sequencing. The metadata of SARS-CoV-2 deposited in SRA (276,799 entries) were downloaded on 18 February 2022 (Supplementary Materials) and classified into three groups (I: <600 bp; II: 600–1600 bp; III: ≥1600 bp) per amplicon length. One thousand sequencing datasets were selected in each group and input into our analytical system. Spike protein variation information of 2349 samples was clearly revealed (Supplementary Table S3), highlighting the robustness of our method. Furthermore, 373, 210, and 68 datasets corresponding to groups I, II, and III, respectively, were indicated as ‘multiple-fragment consensus’ or ‘segment-missing amplicon.’ This result again indicated that the rate of successful sequencing increased with the length of amplicon, which was negatively associated with the number of primers. Figure 3 presents the data count given with the <25-read sequencing depth across the spike gene. Obviously, the count yielded in group I was generally higher than that in group II, followed by that in group III. This again indicates that the higher the number of primers, the lower the sequencing depth and the higher the number of missing amplicons, presumably due to unsatisfactory hybridisation efficiency of the primers with the mutated sequence of SARS-CoV-2. Thus, the protocol using primer set IV can unravel mutation information and can be integrated with our protocol to avoid underestimating mutated sequences by examining low-depth sequencing fragments.

## 3. Discussion

By taking advantage of the in silico evaluation of primer sets for spike gene sequences of SARS-CoV-2, we have proposed a single-tube PCR containing only five oligo primers, which can be easily modified for new variants. We also proposed a bioinformatic protocol that can process FASTQ reads into spike gene consensus sequences. Our protocol has three advantages over several currently available methods.

### 3.1. A General Method Requiring No Primer Information

Different primer sets have been applied to generate SARS-CoV-2 genomes (Table 1). In the typical ARTIC protocol (18), a scheme.bed file is necessarily input to detail the user-designed primer (see Supplementary Materials for details). Our analytical protocol works well with the data yielded from versatile designs of primers, thus facilitating easy determination of spike protein variants.

### 3.2. Resolve the Misrepresentation of Mutation Caused by Unknown Nucleotide (N)

A comparison between the analytical results of the PRJNA675364 sequencing data [15] using the ARTIC and our protocol revealed an inconsistent variant calling in 5 of 157 samples (Supplementary Table S1). An unknown nucleotide ‘N’ was reported in the consensus sequences of these five samples produced using the ARTIC protocol (Supplementary Table S2), leading to absent variant calling at SRR13021047 (D614G), SRR13021061 (D830Y and G1246A), SRR13021137 (D578Y), and SRR13021139 (D614G) (see Supplementary Materials for details). By contrast, our bioinformatics protocol presented these four variant callings accurately when closely examining read alignments. The fifth inconsistent variant calling was a 29-bp deletion in the spike gene of the SRR13021093 sample. However, our protocol reported a consensus sequence containing this deletion sequence, which suggested that the deletion was not a dominant variant; it was present in only 30% reads, indicating that the ARTIC protocol may overestimate the SARS-CoV-2 variant.

### 3.3. Output Segment-Missing Amplicon to Highlight Inefficient Hybridisation of Primers

Although the shorter amplicons (e.g., primer set I) were thought to benefit the amplification of the highly degraded viral RNA, many amplifications may fail due to the inefficient hybridisation of primers, which results in gaps in the consensus genome. This behaviour occurred in the analysis of B.1.351 variants (aka Beta), in which only 28 of 64 samples (PRJNA694014 [9]) were reported to contain the deletion of amino acid 242–244 owing to the inefficient amplification activated by primer 74 in ARTIC V3 primers. Instead of calling ‘no variant’ or miscalling, our bioinformatic protocol uses ‘segment-missing amplicon’ to present the fragments with low amplification efficiency in the sequencing data. The segment-missing phenomena were frequently observed when applying the small amplicon sequencing approach (ARTIC V3) to Delta variants due to the deletion of amino acids 156–157 [22,38]. The 72R primer of ARTIC V3 was confirmed to be affected by the mutation of the Delta variant. Therefore, ARTIC V4 primers were proposed in June 2021 to replace V3. However, with the viruses mutating frequently, ARTIC V4.1 primers were recently updated for the Omicron variant in December 2021. Furthermore, such segment-missing amplicons resulted in mutation miscalling using the ARTIC protocol followed by the Nextclade (as shown in Supplementary Materials). For example, the deletions of three amino acids at positions 242–244 [9] were miscalled at sites of 243–245 in many samples. 

In the present study, we evaluated the sequencing reads from three amplicon libraries (AvgSpotLen: <600, 600–1600, and ≥1600 bp) and noted that many sequencing data (239,455/276,799 = 86.5%) were derived from short amplicons (<600 bp); however, approximately one quarter of samples in the first set (as illustrated by Figure 3) using the small amplicon tiling protocol (i.e., ARTIC nCoV-2019, set I) provided insufficient reads, precluding accurate reporting of spike gene mutations. Thus, the long amplicon protocol appears to be effective in detecting spike variants. Our observation was also supported by a recent publication [39]. Brejova et al. compared the sequencing results obtained from the libraries containing SARS-CoV-2 samples made of 400 bp (primer set I), 2000 bp, and 2500 bp (primer set III) amplicon pools to conclude that sequencing long amplicons clearly outperforms shorter amplicons in terms of lower coverage variation and overall quality of the consensus sequences.

This study also had some limitations. Although our one-base-overlapping primers can be conducted in a single PCR, the sensitivity of detecting clinical samples was not systematically examined. A study encompassing viral loads at a broad range is preferred rather than the present study that contains six clinical samples in connection with limited Ct ranges (13.8–23.7). Our bioinformatic pipeline provides consensus variants of spike proteins and does not report intra-host diversity. Nevertheless, this protocol provides an advanced approach enabling future studies to create a surveillance system and monitor spike gene variants of SARS-CoV-2 continuously.

## 4. Materials and Methods

### 4.1. In Silico Evaluation of Primer Sets

Multiplex primers have been designed for the targeted amplification of the SARS-CoV-2 genome (Table 1) [15,19,21,31,32]. The primer sequences were downloaded and analysed in silico against complete SARS-CoV-2 genome sequences deposited in the NCBI database (14 February 2022). A PCR amplicon was in silico detected as a pair of primer exact matches to a reference genome with the desired amplicon size using the SeqKit toolkit [40]. The detection rate of each primer pair was therefore estimated by dividing the in silico PCR product number by the total number of genome sequences (848,003). To calculate the detection rates of primer sets for the complete genome and the spike gene of SARS-CoV-2, the number of sequences containing all primer pairs except for the first and end primer pairs and the number of sequences containing the primer pairs covering spike genes were used, respectively.

### 4.2. Primer Design and Spike Gene Variation

More than 80,000 genome sequences were downloaded from GISAID on 15 August 2020. Sequences containing more than five Ns were removed, the remaining sequences were reformatted to upper case, and ‘U’ was replaced with ‘T,’ resulting in 29,552 complete sequences. Two primer pairs with one-base overlapping were designed—S1F: GGCATAATGATGAATGTCGCAA, S1R: AACCATTGAAGTTGAAATTGACACA, S2F: TTAACAGGCACAGGTGTTCTTACTG, and S2R: TTGACTCCTTTGAGCACTGGC—to have an annealing temperature range from 65.4 to 67.0 °C, as predicted by the Multiple Primer Analyzer (Thermo Fisher Scientific, Waltham, MA, USA), and to in silico-produce desired amplicons from 29,386 sequences (detection rate = 99.44%). This primer set was later evaluated against the 848,003 genome sequences obtained from NCBI (14 February 2022), and primer sequence TTAAAAGGCACAGGTGTTCTTACTG was added to S2F to reach the detection rate of 97.07% for in silico-generating amplicons spanning from 20,794 to 25,347 of the Wuhan-Hu-1 reference genome (MN908947.3). To monitor spike protein variations, the mutation table based on 7,919,209 sequences was downloaded from the hCov19 Mutation Dashboard on 12 February 2022 [33]. Mutation occurrences greater than 1000 among the 7,919,209 sequences were retrieved to obtain 446 variant sites. The mutation rates of the spike protein were estimated based on mutation occurrence at the specific amino acid position (Figure 2).

### 4.3. Single PCR and Nanopore Sequencing

SARS-CoV-2 was obtained from the Taiwan Centers for Disease Control. The virus was amplified as described previously [41]. Viral RNA was extracted from the cultured supernatant using the QIAzol Lysis Reagent (Qiagen, Germantown, MD, USA) following the manufacturer’s instructions. Reverse transcription was conducted using random primers and Superscript IV transcriptase (Invitrogen, Vilnius, Lithuania), following the manufacturer’s instructions. A single PCR was achieved using the platinum SuperFi II green PCR master mix (Invitrogen). The PCR mix included 25 µL of PCR master mix, 5 µL of 10 µM primer mix (S1F, S1R, S2F, and S2R mixed evenly), 2 µL of cDNA, and 18 µL of nuclease-free water. The PCR programme was set for an initial denaturation at 98 °C for 30 s, 35 cycles of denaturation at 98 °C for 10 s, annealing at 60 °C for 10 s and extension at 72 °C for 1 min, and a final extension at 72 °C for 5 min. PCR amplicon clean-up was conducted using an equal volume of AMPure XP (Beckman Coulter, Brea, CA, USA), following the manufacturer’s instructions. The sequencing library was constructed using a Rapid Barcoding Kit (Item# SQK-RBK004, Oxford Nanopore Technologies, Oxford, UK). Briefly, for each barcode, 400 ng of DNA in 7.5 µL was mixed with 2.5 µL of one rapid barcode and incubated at 30 °C for 1 min and then at 80 °C for 1 min. All barcoded samples were pooled in one tube, and an equal volume of AMPure XP was added for clean-up. DNA was eluted with 10 µL of 10 mM Tris-HCl pH 7.5–8.0 with 50 mM NaCl; then, 1 µL of rapid adaptor was added, followed by a 10 min incubation at room temperature. The presequencing mix, 34 µL of sequencing buffer, 25.5 µL of loading beads, 4.5 µL of nuclease-free water, and 11 µL of DNA library, was loaded to a flowcell for sequencing. Real-time basecalling and demultiplexing were performed using Guppy v4.2.2 in MinKNOW v20.10.3 to produce FASTQ files of samples.

### 4.4. Clinical Samples

Six SARS-CoV-2 cDNAs were obtained from the Kaohsiung Veterans General Hospital. cDNA synthesis was performed on the extracted RNA using LunaScript® RT SuperMix Kit (cat# M3010, New England BioLabs, Ipswich, MA, USA) followed by our proposed multiplex PCR using 12.5 µL of PCR master mix, 2.5 µL of 10 µM primer mix (S1F, S1R, S2F, and S2R mixed evenly), 2 µL of cDNA, and 8 µL of nuclease-free water to cover the spike gene. PCR products were used directly without further cleanup for library construction using Rapid Barcoding Kit 96 (Item# SQK-RBK110.96, Oxford Nanopore Technologies, Oxford, UK): a mixture of 5 µL of PCR product, 2.5 µL of nuclease-free water, and 2.5 µL of one rapid barcode was incubated at 30 °C for 2 min and then at 80 °C for 2 min. All barcoded samples were pooled in one tube, and an equal volume of Solid Phase Reversible Immobilization beads was added for clean-up. An aliquot of 800 ng of barcoded DNA was used to make up a total volume of 11 µL with EB. One microliter of rapid adaptor was added, followed by 10 min of incubation at room temperature. The presequencing mix was prepared by adding 37.5 µL of Sequencing Buffer II and 25.5 µL of loading beads to a 12 µL DNA library.

### 4.5. Bioinformatic Analysis

Customised scripts written in Python are available at https://github.com/jade-nhri/covid19S (accessed on 3 November 2021). In downloadSRA.py, the BioProject accession was used to retrieve the metadata, and the corresponding sequencing runs in FASTQ were downloaded parallelly using fastq-dump in the SRA Toolkit (v2.11.0). To demonstrate our bioinformatic protocol, three BioProject sequencing datasets—PRJNA694014 [9], PRJNA645718 [21], and PRJNA675364 [15] (Table 1)—were downloaded using downloadSRA.py, which contain 64, 5, and 157 FASTQ files, respectively. Using runtrimming.py, sequencing was trimmed parallelly from the termini of reads with the SeqKit toolkit [40]. In runconsensus.py, the FASTQ files of each sample were separately aligned to the spike gene (MN908947.3: 21563-25384) using Medaka v1.2.6 (medaka_consensus with -g -f parameters) iteratively to produce consensus spike genes. The -g parameter in medaka_consensus did not fill gaps in consensus generation; therefore, multiple consensus sequences were produced if insufficient sequencing reads were present in some positions. If the subsequent consensus sequence was identical to the preceding sequence, the filename of the subsequent sequence was renamed and suffixed with ‘_final.fa.’ The final consensus file of each sample was concatenated to form consensus.fasta if it contained a single sequence with a length more than 3000 bp; the samples were otherwise labelled as ‘multiple-fragment consensus.’ A file containing consensus sequences of spike genes was uploaded to Nextclade (v0.14.2) for clade assignment and variant calling [42]. In getvar.py, the consensus spike genes were first aligned to the spike gene for homopolymer correction [43], and the corrected sequences were then translated to compare with the spike protein (QHD43416.1) for variant calling. Three forward frames were used to translate nucleotides into their corresponding amino acid sequences. If a sequence with more than two stop codons in its translated sequence was detected, its alignment file (calls_to_draft.bam) was further examined using pysam, and an ambiguous nucleotide ‘N’ was used to substitute the position with insufficient sequencing depth (<50). If multiple sequences or a single short sequence (<2000 bp) was obtained after splitting at ‘N,’ the samples were labelled as ‘segment-missing amplicon.’ Finally, spike protein variants, nucleotides, and amino acid sequences of each sample were summarised in a file named Result.csv.

## Figures and Tables

**Table 1:** Basic descriptions and primer evaluations of amplicon tiling methods for SARS-CoV-2 genomes.

Name	ARTIC nCoV-2019 V4, Primer Set I	SARS-Cov-2-Midnight, Primer Set II	14 × 2.5 kb Amplicons, Primer Set III
Amplicon length	400 bp	1200 bp	2500 bp
Primer pairs	99	29	14
Protocol/Reference	https://www.protocols.io/view/ncov-2019-sequencing-protocol-v3-locost-bh42j8ye (accessed on 14 February 2022) [19]	https://www.protocols.io/view/sars-cov2-genome-sequencing-protocol-1200bp-amplic-btsrnnd6 (accessed on 14 February 2022) [31]	[15,32]
Primer sequences	https://github.com/artic-network/artic-ncov2019/blob/master/primer_schemes/nCoV-2019/V4/SARS-CoV-2.primer.bed (accessed on 14 February 2022)	https://www.protocols.io/view/sars-cov2-genome-sequencing-protocol-1200bp-amplic-btsrnnd6?step=4.1 (accessed on 14 February 2022) [21]	[15,32]
Projects in SRA	PRJEB37886 [27], PRJEB41737 [16], PRJNA694014 [9]	PRJNA645718 [21]	PRJNA675364 [15]
Percentage of genome containing all primer pairs (%)	V4: 9.58; V4.1: 14.53 (spanning from 344 to 29,512)	28.30 (spanning from 1128 to 29,790)	58.53 (spanning from 1897 to 28,145)
Primer covering spike gene	71–84	22–25	A6 and B6
Percentage of genome containing spike gene primers (%)	V4: 57.87 and V4.1: 59.49 (spanning from 21,316 to 25,438)	76.19 (spanning from 21,562 to 25,790)	92.20 (spanning from 21,386 to 25,646)

## Data Availability

The bioinformatic protocol is openly available on Github (https://github.com/jade-nhri/covid19S). Supplementary Materials available at https://doi.org/10.6084/m9.figshare.16929472. Supplementary Figures available at https://doi.org/10.6084/m9.figshare.16929484. Supplementary Table S1 available at https://doi.org/10.6084/m9.figshare.16929445. Supplementary Table S2 available at https://doi.org/10.6084/m9.figshare.16929448. Supplementary Table S3 available at https://doi.org/10.6084/m9.figshare.16929463.

## Supplementary Materials

The following are available online at https://www.mdpi.com/article/10.3390/ijms23063257/s1.
