# Supplementary material for: High-Integrity Sequencing of Spike Gene for SARS-CoV-2 Variant Determination
Source: Int J Mol Sci. 2022 Mar 17;23(6):3257. doi: 10.3390/ijms23063257 (PMC8954144; doi:10.3390/ijms23063257)
Supplement: Supplementary file 1 [file ijms-23-03257-s001.zip › Supplementary_Data.pdf]

Supplementary Data

Covid19S: https://github.com/jade-nhri/covid19S

cd ~

git clone https://github.com/jade-nhri/covid19S.git

cd covid19S

docker build -t "covids_202105:v1" ./

docker run -h covidS --name covidS -i -t -v /:/MyData covids_202105:v1 /bin/bash

root@covidS:/#

Please note that you need to run “vdb-config --interactive” before using sra toolkit.

A quick test run for PRJNA645718 (10 minutes):

Inside covidS container:

root@covidS:/opt# cd ~

root@covidS:~# mkdir Run

root@covidS:~# cd Run/

root@covidS:~/Run# downloadSRA.py -o PRJNA645718 --project PRJNA645718

```
root@covidS:~/Run# downloadSRA.py -o PRJNA645718 --project PRJNA645718
wget 'http://trace.ncbi.nlm.nih.gov/Traces/sra/sra.cgi?save=efetch&rettype=runinfo&db=sra&term=PRJNA645718' -O - > sralist.csv
['OXFORD_NANOPORE']
OXFORD_NANOPORE
          Run          ReleaseDate             LoadDate   spots     bases  ...  Submission  dbgap_study_accession  Consent                          RunHash                          ReadHash
0  SRR12209729  2020-07-13 12:02:15  2020-07-13 11:58:53  114879  58446889  ...  SRA1098098                    NaN   public  94D766BE3DA824466D6D3105C9D416CF  7F419F3C494C7B222ADFAB84A2014704
1  SRR12209728  2020-07-13 12:02:15  2020-07-13 11:58:44   58899  26918002  ...  SRA1098098                    NaN   public  877E9230B2C290D675A46A1A3DDDCEF4  10E36658F3BCA50152D402461877AF39
2  SRR12209727  2020-07-13 12:02:15  2020-07-13 11:58:49   56842  26118431  ...  SRA1098098                    NaN   public  4005EDCC86DC05168D363E0EDC0D2F68  D7FB53F83CC5BC2722843E6C5B90AC89
3  SRR12209726  2020-07-13 12:02:15  2020-07-13 11:58:47   53001  25871724  ...  SRA1098098                    NaN   public  8E3B7ACB46FFFAFC9CDBE26584BF35D4  9B37FE32C1DBF2B9E6E6B51A4EB4772B
4  SRR12209725  2020-07-13 12:02:15  2020-07-13 11:58:50   45168  22086531  ...  SRA1098098                    NaN   public  9E65D4CA7954C592597F2C37C55D59C6  684616AB3F5E1CDD0DD332DC23D2344E

[5 rows x 47 columns]
          Run          ReleaseDate             LoadDate   spots     bases  ...  Submission  dbgap_study_accession  Consent                          RunHash                          ReadHash
0  SRR12209729  2020-07-13 12:02:15  2020-07-13 11:58:53  114879  58446889  ...  SRA1098098                    NaN   public  94D766BE3DA824466D6D3105C9D416CF  7F419F3C494C7B222ADFAB84A2014704
1  SRR12209728  2020-07-13 12:02:15  2020-07-13 11:58:44   58899  26918002  ...  SRA1098098                    NaN   public  877E9230B2C290D675A46A1A3DDDCEF4  10E36658F3BCA50152D402461877AF39
2  SRR12209727  2020-07-13 12:02:15  2020-07-13 11:58:49   56842  26118431  ...  SRA1098098                    NaN   public  4005EDCC86DC05168D363E0EDC0D2F68  D7FB53F83CC5BC2722843E6C5B90AC89
3  SRR12209726  2020-07-13 12:02:15  2020-07-13 11:58:47   53001  25871724  ...  SRA1098098                    NaN   public  8E3B7ACB46FFFAFC9CDBE26584BF35D4  9B37FE32C1DBF2B9E6E6B51A4EB4772B
4  SRR12209725  2020-07-13 12:02:15  2020-07-13 11:58:50   45168  22086531  ...  SRA1098098                    NaN   public  9E65D4CA7954C592597F2C37C55D59C6  684616AB3F5E1CDD0DD332DC23D2344E

[5 rows x 47 columns]
['SRR12209729' 'SRR12209728' 'SRR12209727' 'SRR12209726' 'SRR12209725']
fastq-dump SRR12209729
fastq-dump SRR12209728
fastq-dump SRR12209727
fastq-dump SRR12209726
fastq-dump SRR12209725
```

```
root@covidS:~/Run# cd PRJNA645718/
root@covidS:~/Run/PRJNA645718# ll
total 335096
drwxr-xr-x 2 root root      4096 May 14 12:18 ./
drwxr-xr-x 3 root root      4096 May 14 12:18 ../
-rw-r--r-- 1 root root  47478886 May 14 12:18 SRR12209725.fastq
-rw-r--r-- 1 root root  55631076 May 14 12:19 SRR12209726.fastq
-rw-r--r-- 1 root root  56406196 May 14 12:19 SRR12209727.fastq
-rw-r--r-- 1 root root  58159154 May 14 12:19 SRR12209728.fastq
-rw-r--r-- 1 root root 125430182 May 14 12:19 SRR12209729.fastq
-rw-r--r-- 1 root root      2982 May 14 12:18 sralist.csv
```

root@covidS:~/Run# runconsensus.py -i PRJNA645718/ -o PRJNA645718/01_consensus

```
root@covidS:~/Run# grep '>' PRJNA645718/01_consensus/consensus.fasta
>SRR12209725
>SRR12209726
>SRR12209727
>SRR12209728
>SRR12209729
```

```
root@covidS:~/Run# ll PRJNA645718/01_consensus/*_final.fa
-rw-r--r-- 1 root root 3856 May 14 12:21 PRJNA645718/01_consensus/SRR12209725_final.fa
-rw-r--r-- 1 root root 3856 May 14 12:22 PRJNA645718/01_consensus/SRR12209726_final.fa
-rw-r--r-- 1 root root 3856 May 14 12:23 PRJNA645718/01_consensus/SRR12209727_final.fa
-rw-r--r-- 1 root root 3856 May 14 12:24 PRJNA645718/01_consensus/SRR12209728_final.fa
-rw-r--r-- 1 root root 3856 May 14 12:25 PRJNA645718/01_consensus/SRR12209729_final.fa
```

root@covidS:~/Run# getvar.py -i PRJNA645718/01_consensus/ -o PRJNA645718/02_output -q PRJNA645718/

```
root@covidS:~/Run# ll PRJNA645718/02_output/
total 60
drwxr-xr-x 2 root root  4096 May 14 12:27 ./
drwxr-xr-x 4 root root  4096 May 14 12:27 ../
-rw-r--r-- 1 root root 25591 May 14 12:27 Result.csv
-rw-r--r-- 1 root root 19180 May 14 12:27 output.fasta
-rw-r--r-- 1 root root    81 May 14 12:27 output.txt
root@covidS:~/Run# cat PRJNA645718/02_output/Result.csv
```

Result.csv:

Name	Variants	NT	AA
SRR12209725		ATGTTTGTTTTTCTTGTTTTAT	MFVFLVLLPLVSSQCVNLTTRTQLPPAYTNS
SRR12209726		ATGTTTGTTTTTCTTGTTTTAT	MFVFLVLLPLVSSQCVNLTTRTQLPPAYTNS
SRR12209727	D614G;A846V	ATGTTTGTTTTTCTTGTTTTAT	MFVFLVLLPLVSSQCVNLTTRTQLPPAYTNS
SRR12209728		ATGTTTGTTTTTCTTGTTTTAT	MFVFLVLLPLVSSQCVNLTTRTQLPPAYTNS
SRR12209729	D614G	ATGTTTGTTTTTCTTGTTTTAT	MFVFLVLLPLVSSQCVNLTTRTQLPPAYTNS

To run covid19S on dilution samples

A sample of 10^-4 of viral RNA (Ct=28.07)

runconsensus.py -i fastq/ -o BC20_consensus -s BC20

getvar.py -i BC20_consensus/ -o BC20_output

cat BC20_output/output.txt

BC20 I68-;H69-;V70-;S71-;G72-;T73-;N74-;G75-;T76-

A sample of 10^-5 of viral RNA (Ct=31.79)

runconsensus.py -i fastq/ -o BC59_consensus -s BC59

"BC59 was not included in the consensus.fasta" was shown in the screen.

Two consensus sequences were produced:

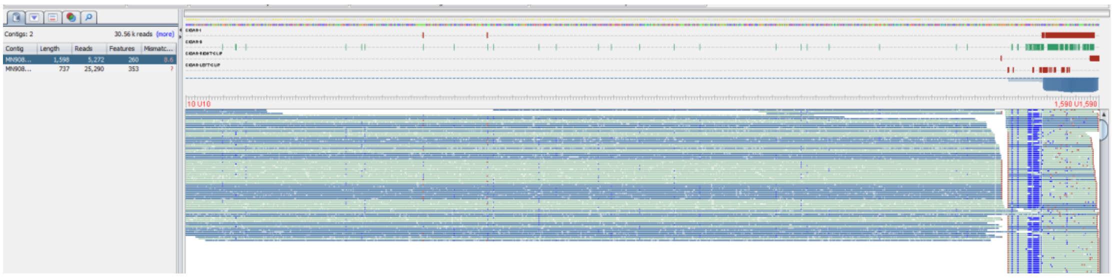

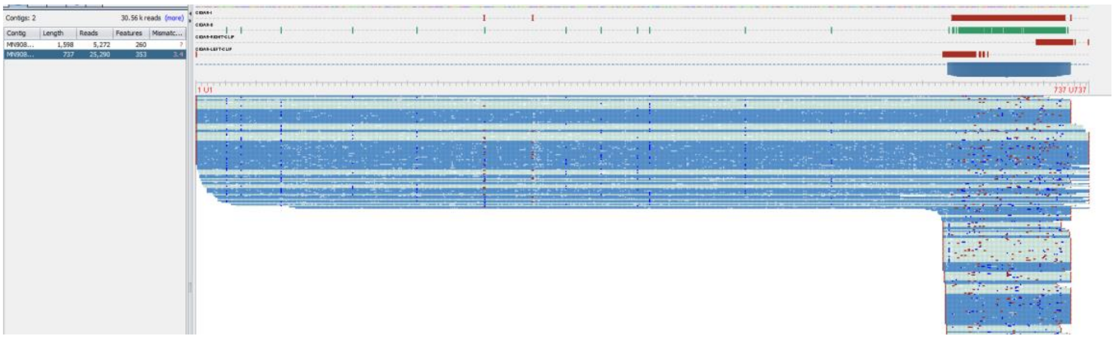

In order to show the read profile, medaka_consensus was performed:

medaka_consensus -i BC59.fastq -d /opt/covid19S/covid19S/Sgene.fasta -g

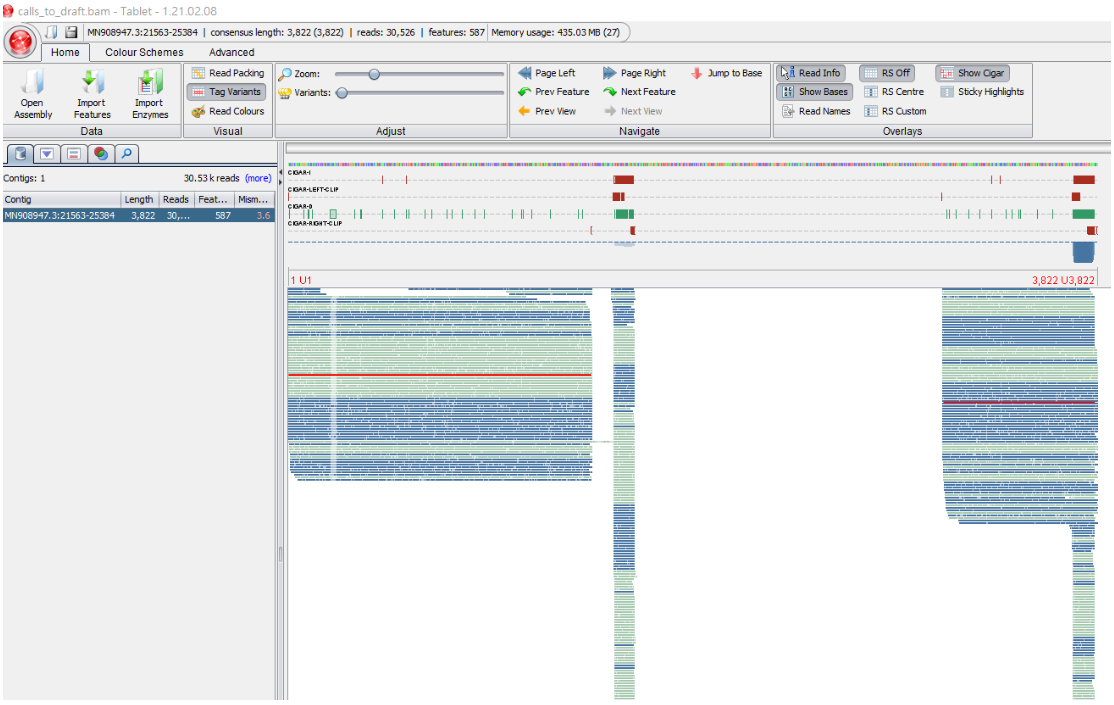

This alignment (Supplementary Figure S1) shows that partial amplicon sequences of spike gene (i.e. "multiple-fragment consensus") were obtained using our primer set. To demonstrate that those reads were part of SARS-CoV-2 genome, two sequences were arbitrary selected for BLAST against Nucleotide collection (nr/nt):

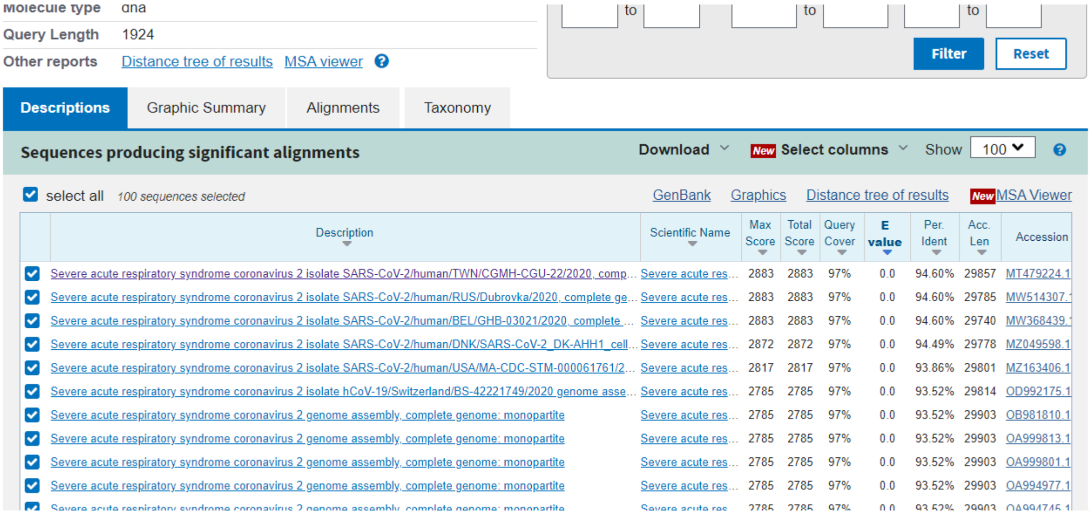

Query Length 1924
Other reports Distance tree of results MSA viewer

Descriptions | Graphic Summary | Alignments | Taxonomy

Sequences producing significant alignments Download Select columns Show 100

select all 100 sequences selected GenBank Graphics Distance tree of results MSA Viewer

Description	Scientific Name	Max Score	Total Score	Query Cover	E value	Per. Ident	Acc. Len	Accession
Severe acute respiratory syndrome coronavirus 2 isolate SARS-CoV-2/human/TWN/CGMH-CGU-22/2020, comp...	Severe acute res...	2883	2883	97%	0.0	94.60%	29857	MT479224.1
Severe acute respiratory syndrome coronavirus 2 isolate SARS-CoV-2/human/RUS/Dubrovka/2020, complete ge...	Severe acute res...	2883	2883	97%	0.0	94.60%	29785	MW514307.
Severe acute respiratory syndrome coronavirus 2 isolate SARS-CoV-2/human/BEL/GHB-03021/2020, complete ...	Severe acute res...	2883	2883	97%	0.0	94.60%	29740	MW368439.
Severe acute respiratory syndrome coronavirus 2 isolate SARS-CoV-2/human/DNK/SARS-CoV-2_DK-AHH1_cell...	Severe acute res...	2872	2872	97%	0.0	94.49%	29778	MZ049598.1
Severe acute respiratory syndrome coronavirus 2 isolate SARS-CoV-2/human/USA/MA-CDC-STM-000061761/2...	Severe acute res...	2817	2817	97%	0.0	93.86%	29801	MZ163406.1
Severe acute respiratory syndrome coronavirus 2 isolate hCoV-19/Switzerland/BS-42221749/2020 genome asse...	Severe acute res...	2785	2785	97%	0.0	93.52%	29814	OD992175.1
Severe acute respiratory syndrome coronavirus 2 genome assembly, complete genome: monopartite	Severe acute res...	2785	2785	97%	0.0	93.52%	29903	OB981810.1
Severe acute respiratory syndrome coronavirus 2 genome assembly, complete genome: monopartite	Severe acute res...	2785	2785	97%	0.0	93.52%	29903	OA999813.1
Severe acute respiratory syndrome coronavirus 2 genome assembly, complete genome: monopartite	Severe acute res...	2785	2785	97%	0.0	93.52%	29903	OA999801.1
Severe acute respiratory syndrome coronavirus 2 genome assembly, complete genome: monopartite	Severe acute res...	2785	2785	97%	0.0	93.52%	29903	OA994977.1
Severe acute respiratory syndrome coronavirus 2 genome assembly, complete genome: monopartite	Severe acute res...	2785	2785	97%	0.0	93.52%	29903	OA994745.1

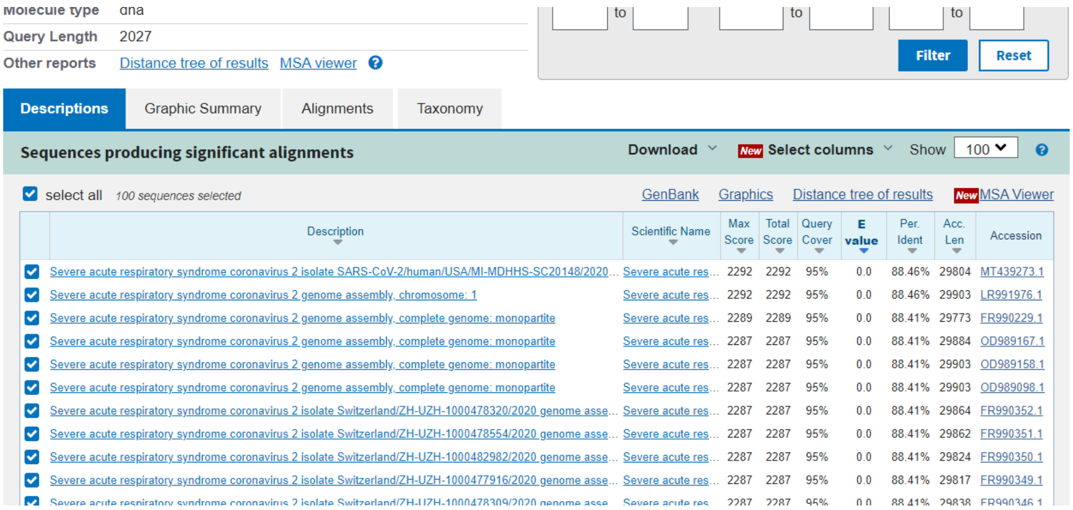

Molecule type	dna
Query Length	2027
Other reports	Distance tree of results MSA viewer

Descriptions | Graphic Summary | Alignments | Taxonomy

Sequences producing significant alignments Download New Select columns Show 100

select all 100 sequences selected GenBank Graphics Distance tree of results New MSA Viewer

Description	Scientific Name	Max Score	Total Score	Query Cover	E value	Per. Ident	Acc. Len	Accession
Severe acute respiratory syndrome coronavirus 2 isolate SARS-CoV-2/human/USA/MI-MDHHS-SC20148/2020...	Severe acute res...	2292	2292	95%	0.0	88.46%	29804	MT439273.1
Severe acute respiratory syndrome coronavirus 2 genome assembly, chromosome: 1	Severe acute res...	2292	2292	95%	0.0	88.46%	29903	LR991976.1
Severe acute respiratory syndrome coronavirus 2 genome assembly, complete genome: monopartite	Severe acute res...	2289	2289	95%	0.0	88.41%	29773	FR990229.1
Severe acute respiratory syndrome coronavirus 2 genome assembly, complete genome: monopartite	Severe acute res...	2287	2287	95%	0.0	88.41%	29884	OD989167.1
Severe acute respiratory syndrome coronavirus 2 genome assembly, complete genome: monopartite	Severe acute res...	2287	2287	95%	0.0	88.41%	29903	OD989158.1
Severe acute respiratory syndrome coronavirus 2 genome assembly, complete genome: monopartite	Severe acute res...	2287	2287	95%	0.0	88.41%	29903	OD989098.1
Severe acute respiratory syndrome coronavirus 2 isolate Switzerland/ZH-UZH-1000478320/2020 genome asse...	Severe acute res...	2287	2287	95%	0.0	88.41%	29864	FR990352.1
Severe acute respiratory syndrome coronavirus 2 isolate Switzerland/ZH-UZH-1000478554/2020 genome asse...	Severe acute res...	2287	2287	95%	0.0	88.41%	29862	FR990351.1
Severe acute respiratory syndrome coronavirus 2 isolate Switzerland/ZH-UZH-1000482982/2020 genome asse...	Severe acute res...	2287	2287	95%	0.0	88.41%	29824	FR990350.1
Severe acute respiratory syndrome coronavirus 2 isolate Switzerland/ZH-UZH-1000477916/2020 genome asse...	Severe acute res...	2287	2287	95%	0.0	88.41%	29817	FR990349.1
Severe acute respiratory syndrome coronavirus 2 isolate Switzerland/ZH-UZH-1000478309/2020 genome asse...	Severe acute res...	2287	2287	95%	0.0	88.41%	29838	FR990346.1

Likewise, similar results were obtained by BLAST the consensus sequences against Nucleotide collection (nr/nt):

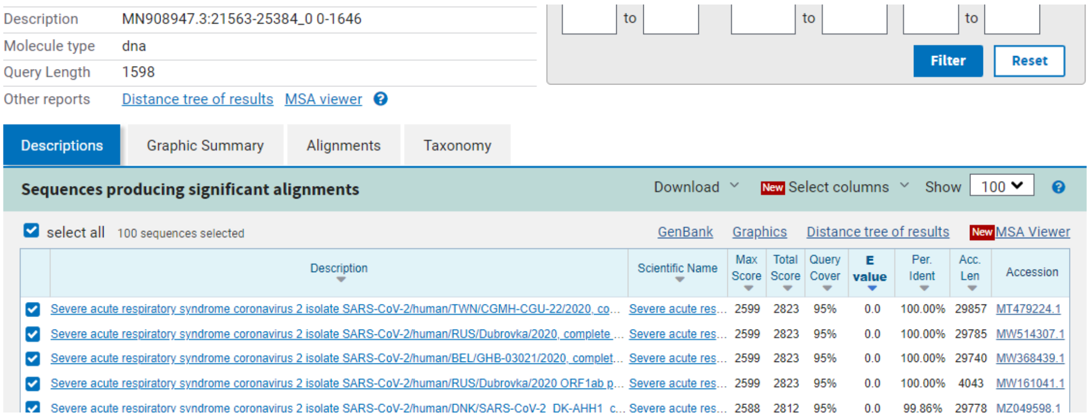

Description	MN908947.3:21563-25384_0 0-1646
Molecule type	dna
Query Length	1598
Other reports	Distance tree of results MSA viewer

Descriptions | Graphic Summary | Alignments | Taxonomy

Sequences producing significant alignments Download New Select columns Show 100

select all 100 sequences selected GenBank Graphics Distance tree of results New MSA Viewer

Description	Scientific Name	Max Score	Total Score	Query Cover	E value	Per. Ident	Acc. Len	Accession
Severe acute respiratory syndrome coronavirus 2 isolate SARS-CoV-2/human/TWN/CGMH-CGU-22/2020, co...	Severe acute res...	2599	2823	95%	0.0	100.00%	29857	MT479224.1
Severe acute respiratory syndrome coronavirus 2 isolate SARS-CoV-2/human/RUS/Dubrovka/2020, complete ...	Severe acute res...	2599	2823	95%	0.0	100.00%	29785	MW514307.1
Severe acute respiratory syndrome coronavirus 2 isolate SARS-CoV-2/human/BEL/GHB-03021/2020, complet...	Severe acute res...	2599	2823	95%	0.0	100.00%	29740	MW368439.1
Severe acute respiratory syndrome coronavirus 2 isolate SARS-CoV-2/human/RUS/Dubrovka/2020 ORF1ab p...	Severe acute res...	2599	2823	95%	0.0	100.00%	4043	MW161041.1
Severe acute respiratory syndrome coronavirus 2 isolate SARS-CoV-2/human/DNK/SARS-CoV-2_DK-AHH1 c...	Severe acute res...	2588	2812	95%	0.0	99.86%	29778	MZ049598.1

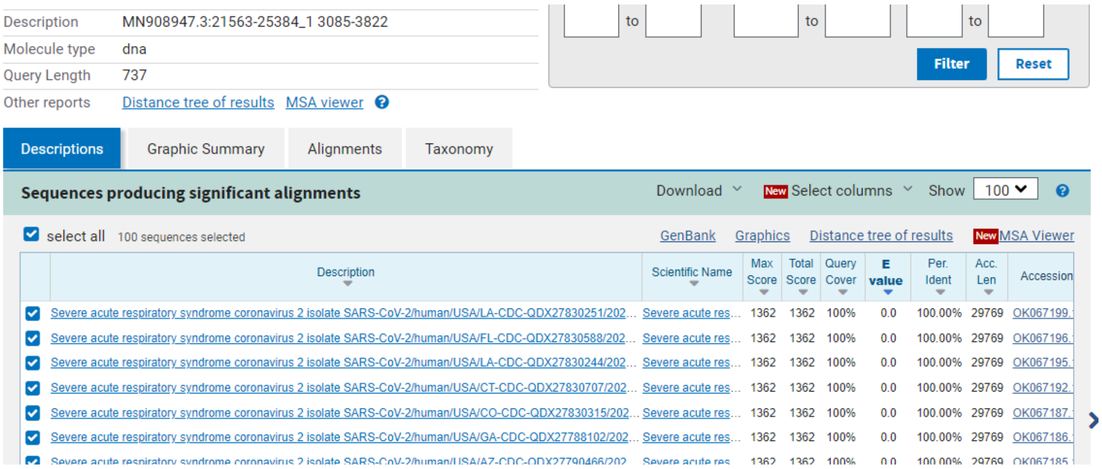

Description	MN908947.3:21563-25384_1 3085-3822
Molecule type	dna
Query Length	737
Other reports	Distance tree of results MSA viewer

Descriptions | Graphic Summary | Alignments | Taxonomy

Sequences producing significant alignments Download New Select columns Show 100

select all 100 sequences selected GenBank Graphics Distance tree of results New MSA Viewer

Description	Scientific Name	Max Score	Total Score	Query Cover	E value	Per. Ident	Acc. Len	Accession
Severe acute respiratory syndrome coronavirus 2 isolate SARS-CoV-2/human/USA/LA-CDC-QDX27830251/202...	Severe acute res...	1362	1362	100%	0.0	100.00%	29769	OK067199.
Severe acute respiratory syndrome coronavirus 2 isolate SARS-CoV-2/human/USA/FL-CDC-QDX27830588/202...	Severe acute res...	1362	1362	100%	0.0	100.00%	29769	OK067196.
Severe acute respiratory syndrome coronavirus 2 isolate SARS-CoV-2/human/USA/LA-CDC-QDX27830244/202...	Severe acute res...	1362	1362	100%	0.0	100.00%	29769	OK067195.
Severe acute respiratory syndrome coronavirus 2 isolate SARS-CoV-2/human/USA/CT-CDC-QDX27830707/202...	Severe acute res...	1362	1362	100%	0.0	100.00%	29769	OK067192.
Severe acute respiratory syndrome coronavirus 2 isolate SARS-CoV-2/human/USA/CO-CDC-QDX27830315/202...	Severe acute res...	1362	1362	100%	0.0	100.00%	29769	OK067187.
Severe acute respiratory syndrome coronavirus 2 isolate SARS-CoV-2/human/USA/GA-CDC-QDX27788102/202...	Severe acute res...	1362	1362	100%	0.0	100.00%	29769	OK067186.
Severe acute respiratory syndrome coronavirus 2 isolate SARS-CoV-2/human/USA/AZ-CDC-QDX27790466/202	Severe acute res	1362	1362	100%	0.0	100.00%	29769	OK067185.

To run covid19S on PRJNA675364

root@covid19:~# mkdir Run

root@covid19:~# cd Run/

PRJNA675364 (https://www.ncbi.nlm.nih.gov/sra/?term=PRJNA675364), PCR amplification of reverse-transcribed SARS-CoV-2 viral RNA with 14 x 2.5kb amplicons, followed by nanopore sequencing (1).

To download sequencing data using downloadSRA.py:

```
usage: download_SRA1.py [-h] [-o O] [-t T] [--project PROJECT] [--nanopore NANOPORE]

optional arguments:
  -h, --help           show this help message and exit
  -o O                 an output folder
  -t T                 threads (default=100)
  --project PROJECT    SRA projcet ID
  --nanopore NANOPORE  only download Nanopore sequences
```

root@covid19:~/Run# downloadSRA.py -o PRJNA675364 --project PRJNA675364

A folder named PRJNA675364 was produced to contain the 157 corresponding FASTQ files.

```
root@covid19:~/Run# cd PRJNA675364/
root@covid19:~/Run/PRJNA675364# ll *.fastq | head
-rw-r--r-- 1 root root  221127068 May 11 15:06 SRR13020989.fastq
-rw-r--r-- 1 root root  352614430 May 11 15:04 SRR13020990.fastq
-rw-r--r-- 1 root root  337942906 May 11 15:01 SRR13020991.fastq
-rw-r--r-- 1 root root  285115914 May 11 15:07 SRR13020992.fastq
-rw-r--r-- 1 root root  392140224 May 11 15:06 SRR13020993.fastq
-rw-r--r-- 1 root root  395338868 May 11 15:04 SRR13020994.fastq
-rw-r--r-- 1 root root  396053738 May 11 15:07 SRR13020995.fastq
-rw-r--r-- 1 root root  189902332 May 11 15:05 SRR13020996.fastq
-rw-r--r-- 1 root root   67536750 May 11 15:03 SRR13020997.fastq
-rw-r--r-- 1 root root  139461908 May 11 15:05 SRR13020998.fastq
root@covid19:~/Run/PRJNA675364# ll *.fastq | wc -l
157
```

To produce consensus sequences for spike gene:

```
usage: runconsensus.py [-h] [-i I] [-o O] [-t T] [-s S] [-m M] [-r R]

optional arguments:
  -h, --help  show this help message and exit
  -i I        an input folder
  -o O        an output folder
  -t T        threads (default=100)
  -s S        specify a sample name
  -m M        min length of consensus (default=3000 bp)
  -r R        the path to the reference sequence
```

root@covid19:~/Run# runconsensus.py -i PRJNA675364/ -o PRJNA675364/01_consensus

root@covid19:~/Run/PRJNA675364/01_consensus# mv consensus.fasta

PRJNA675364_consensus.fasta
root@covid19:~/Run# grep '>' PRJNA675364/01_consensus/consensus.fasta | wc -l
157

The above consensus sequences are listed in Supplementary Table S2 (PRJNA675364_consensus).

To get spike protein variants:

To upload consensus.fasta to Nextclade (https://clades.nextstrain.org/) for clade assignment and variant calling:

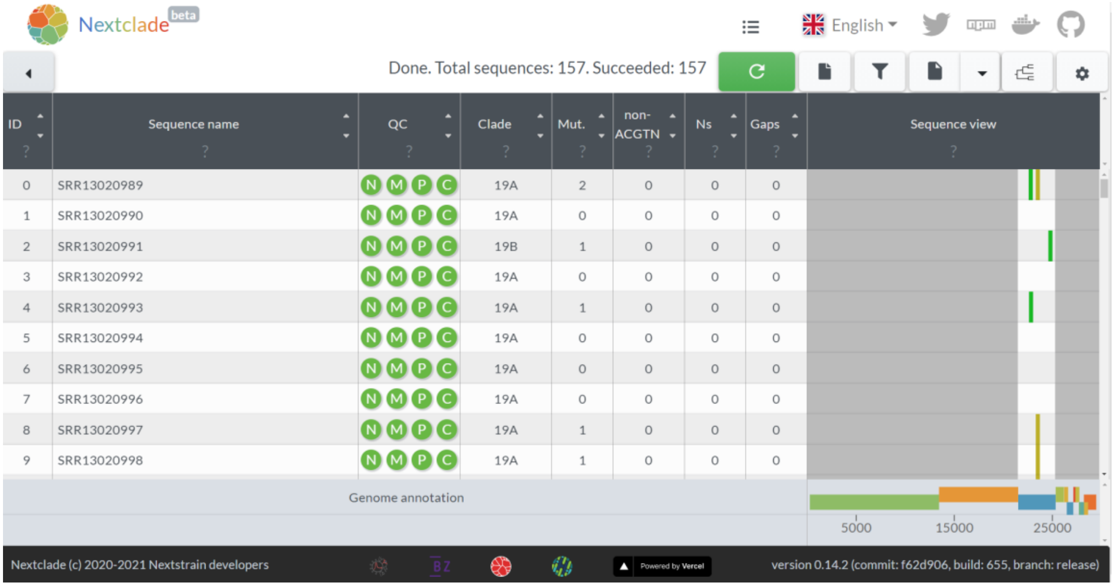

The variants analyzed by Nextclade are listed in Supplementary Table S1 (PRJNA675364's Spike consensus+Nextclade) and the exported TSV results are shown in Supplementary Table S2 (PRJNA675364_Nextclade).

root@covid19:~/Run# getvar.py -i PRJNA675364/01_consensus/ -o PRJNA675364/02_output

```
root@covid19:~/Run# getvar.py -i PRJNA675364/01_consensus/ -o PRJNA675364/02_output/
  gap in subject:AACTTTTACTT==>AACTTTACTT
SRR13021061_final.fa was corrected
```

```
root@covid19:~/Run# cd PRJNA675364/02_output/
root@covid19:~/Run/PRJNA675364/02_output# ll
total 1396
drwxr-xr-x 2 root root   4096 May 12 08:37 ./
drwxr-xr-x 4 root root  12288 May 12 08:37 ../
-rw-r--r-- 1 root root 802805 May 12 08:37 Result.csv
-rw-r--r-- 1 root root 602252 May 12 08:37 output.fasta
-rw-r--r-- 1 root root   2399 May 12 08:37 output.txt
```

root@covid19:~/Run/PRJNA675364/02_output# mv Result.csv PRJNA675364_Result.csv

To trim adapter, barcode and primer sequences using runtrimming.py:

root@covid19:~/Run# runtrimming.py -i PRJNA675364/ -o PRJNA675364_trimmed

```
root@covid19:~/Run# runtrimming.py -i PRJNA675364/ -o PRJNA675364_trimmed
cat /root/Run/PRJNA675364/SRR13020994.fastq | seqkit subseq -r 100:-100 > /root/Run/PRJNA675364_trimmed/SRR13020994.fastq
cat /root/Run/PRJNA675364/SRR13021024.fastq | seqkit subseq -r 100:-100 > /root/Run/PRJNA675364_trimmed/SRR13021024.fastq
cat /root/Run/PRJNA675364/SRR13021122.fastq | seqkit subseq -r 100:-100 > /root/Run/PRJNA675364_trimmed/SRR13021122.fastq
cat /root/Run/PRJNA675364/SRR13021043.fastq | seqkit subseq -r 100:-100 > /root/Run/PRJNA675364_trimmed/SRR13021043.fastq
cat /root/Run/PRJNA675364/SRR13021036.fastq | seqkit subseq -r 100:-100 > /root/Run/PRJNA675364_trimmed/SRR13021036.fastq
cat /root/Run/PRJNA675364/SRR13021034.fastq | seqkit subseq -r 100:-100 > /root/Run/PRJNA675364_trimmed/SRR13021034.fastq
cat /root/Run/PRJNA675364/SRR13021038.fastq | seqkit subseq -r 100:-100 > /root/Run/PRJNA675364_trimmed/SRR13021038.fastq
cat /root/Run/PRJNA675364/SRR13021134.fastq | seqkit subseq -r 100:-100 > /root/Run/PRJNA675364_trimmed/SRR13021134.fastq
cat /root/Run/PRJNA675364/SRR13021089.fastq | seqkit subseq -r 100:-100 > /root/Run/PRJNA675364_trimmed/SRR13021089.fastq
cat /root/Run/PRJNA675364/SRR13021013.fastq | seqkit subseq -r 100:-100 > /root/Run/PRJNA675364_trimmed/SRR13021013.fastq
cat /root/Run/PRJNA675364/SRR13021129.fastq | seqkit subseq -r 100:-100 > /root/Run/PRJNA675364_trimmed/SRR13021129.fastq
cat /root/Run/PRJNA675364/SRR13021090.fastq | seqkit subseq -r 100:-100 > /root/Run/PRJNA675364_trimmed/SRR13021090.fastq
cat /root/Run/PRJNA675364/SRR13021009.fastq | seqkit subseq -r 100:-100 > /root/Run/PRJNA675364_trimmed/SRR13021009.fastq
cat /root/Run/PRJNA675364/SRR13021084.fastq | seqkit subseq -r 100:-100 > /root/Run/PRJNA675364_trimmed/SRR13021084.fastq
cat /root/Run/PRJNA675364/SRR13021066.fastq | seqkit subseq -r 100:-100 > /root/Run/PRJNA675364_trimmed/SRR13021066.fastq
cat /root/Run/PRJNA675364/SRR13021109.fastq | seqkit subseq -r 100:-100 > /root/Run/PRJNA675364_trimmed/SRR13021109.fastq
cat /root/Run/PRJNA675364/SRR13021015.fastq | seqkit subseq -r 100:-100 > /root/Run/PRJNA675364_trimmed/SRR13021015.fastq
```

root@covid19:~/Run# runconsensus.py -i PRJNA675364_trimmed/ -o PRJNA675364_trimmed/01_consensus

root@covid19:~/Run# getvar.py -i PRJNA675364_trimmed/01_consensus/ -o PRJNA675364_trimmed/02_output -q PRJNA675364_trimmed/

 gap in subject:AACTTTTACTT==>AACTTTACTT

SRR13021061_final.fa was corrected

root@covid19:~/Run# grep 'multi' PRJNA675364_trimmed/02_output/Result.csv

root@covid19:~/Run# grep 'segment' PRJNA675364_trimmed/02_output/Result.csv

```
root@covid19:~/Run# runconsensus.py -i PRJNA675364_trimmed/ -o PRJNA675364_trimmed/01_consensus
root@covid19:~/Run# getvar.py -i PRJNA675364_trimmed/01_consensus/ -o PRJNA675364_trimmed/02_output -q PRJNA675364_trimmed/
  gap in subject:AACTTTTACTT==>AACTTTACTT
SRR13021061_final.fa was corrected
```

Please note that there was no difference on variant calling with trimming or not trimming on the PRJNA675364. The results are listed in Supplementary Table S1 (PRJNA675364's Our bioinformatic protocol)

Besides, ARTIC protocol was used to run this project data. According to the bioinformatics protocols (https://artic.network/ncov-2019/ncov2019-bioinformatics-sop.html, https://github.com/Psy-Fer/SARS-CoV-2_GTG (1)), the following commands were used.

git clone https://github.com/artic-network/artic-ncov2019.git

cd artic-ncov2019

conda env remove -n artic-ncov2019

conda env create -f environment.yml

conda activate artic-ncov2019

git clone https://github.com/Psy-Fer/SARS-CoV-2_GTG.git

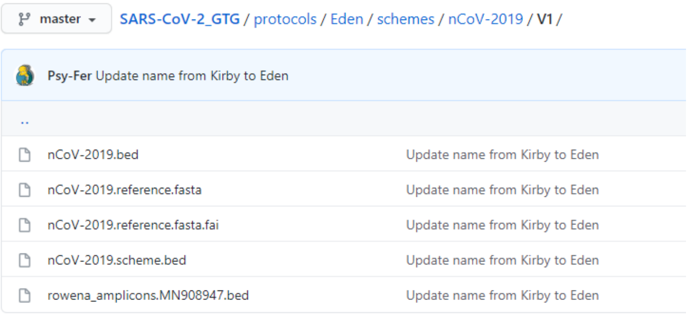

mkdir PRJNA675364

cd PRJNA675364

artic minion --medaka --normalise 200 --threads 100 --scheme-directory path-to-the-schemes-folder --read-file path-to-the-fastq-file nCoV-2019/V1 filename

ll SRR*.consensus.fasta | wc -l

157

cat SRR*.consensus.fasta > PRJNA675364.consensus.fasta

seqkit seq PRJNA675364.consensus.fasta -w 0 > PRJNA675364.ARTIC.consensus.fasta

The consensus sequences are shown in Supplementary Table S2 (PRJNA675364_ARTIC_consensus).

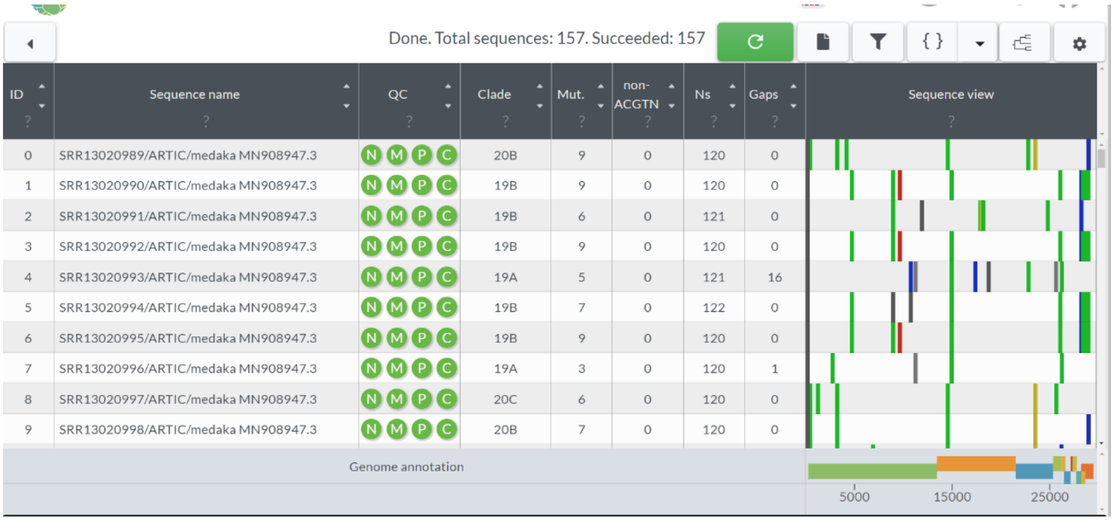

The result analyzed by Nextclade was exported to a TSV file (Supplementary Table S2, PRJNA675364_ARTIC_Nextclade). The spike protein variants are listed in Supplementary Table S1 (PRJNA675364's ARTIC+Nextclade). To compare the results produced by ARTIC and our protocol, five inconsistent variants are in samples of SRR13021047. SRR13021061, SRR13021093, SRR13021137, and SRR13021139.

Our Bioinformatic protocol		ARTIC+Nextclade			
Name	Variants	seqName	clade	aaSubstitutions	aaDeletions
SRR13021047	D614G	SRR13021047/ARTIC/medaka MN908947.3	19A		
SRR13021061	D830Y;G1246A	SRR13021061/ARTIC/medaka MN908947.3	19B		
SRR13021093	E1207D	SRR13021093/ARTIC/medaka MN908947.3	19B	S:Y674X,S:E1207D	S:P665-,S:I666-,S:G667-,S:A668-,S:G669-,S:I670-,S:C671-,S:A672-,S:S673-
SRR13021137	D578Y;D614G	SRR13021137/ARTIC/medaka MN908947.3	20A	S:D614G	
SRR13021139	D614G	SRR13021139/ARTIC/medaka MN908947.3	19A		

In SRR13021047:

Our consensus sequence against the spike gene (MN908947.3:21563-25384)

```
Query  1801  GGAACAAATACTTCTAACCAGGTTGCTGTTCTTTATCAGGGTGTTAACTGCACAGAAGTC  1860
             |||||||||||||||||||||||||||||||||||||||| |||||||||||||||||||
Sbjct  1801  GGAACAAATACTTCTAACCAGGTTGCTGTTCTTTATCAGGATGTTAACTGCACAGAAGTC  1860
```

ARTIC's consensus against the spike gene:

```
Query  23363  GGAACAAATACTTCTAACCAGGTTGCTGTTCTTTATCAGGNTGTTAACTGCACAGAAGTC  23422
              |||||||||||||||||||||||||||||||||||||||| |||||||||||||||||||
Sbjct  1801   GGAACAAATACTTCTAACCAGGTTGCTGTTCTTTATCAGGATGTTAACTGCACAGAAGTC  1860
```

In SRR13021061:

Our consensus sequence against the spike gene:

```
Query  661   TCGGCTTTAGAACCATTGGTAGATTTGCCAATAGGTATTAACATCACTAGGTTTCAAACT  720
             ||||||||||||||||||||||||||||||||||||||||||||||||||||||||||| 
Sbjct  661   TCGGCTTTAGAACCATTGGTAGATTTGCCAATAGGTATTAACATCACTAGGTTTCAAAC-  719

Query  2461  TCTACTTTTCAACAAAGTGACACTTGCATATGCTGGCTTCATCAAACAATATGGTGATTG  2520
             |||||||||||||||||||||||||||| |||||||||||||||||||||||||||||||
Sbjct  2460  TCTACTTTTCAACAAAGTGACACTTGCAGATGCTGGCTTCATCAAACAATATGGTGATTG  2519

Query  3121  TGATTTTTGTGGTAAGGGCTATCATCTTATGTCCTTCCCTCAGTCAGCACCTCATGGTGT  3180
             |||||||||||| |||||||||||||||||||||||||||||||||||||||||||||||
Sbjct  3120  TGATTTTTGTGGAAAGGGCTATCATCTTATGTCCTTCCCTCAGTCAGCACCTCATGGTGT  3179

Query  3721  CTGTAGTTGTCTCAAGGCCTGTTGTTCTTGTGGATCCTGCTGCAAATTTGATGAAGACGA  3780
             ||||||||||||||||| ||||||||||||||||||||||||||||||||||||||||||
Sbjct  3720  CTGTAGTTGTCTCAAGGGCTGTTGTTCTTGTGGATCCTGCTGCAAATTTGATGAAGACGA  3779
```

ARTIC's consensus against the spike gene:

```
Query  22223  TCGGCTTTAGAACCATTGGTAGATTTGCCAATAGGTATTAACATCACTAGGTTTCAAACT  22282
              ||||||||||||||||||||||||||||||||||||||||||||||||||||||||||| 
Sbjct  661    TCGGCTTTAGAACCATTGGTAGATTTGCCAATAGGTATTAACATCACTAGGTTTCAAAC-  719

Query  24023  TCTACTTTTCAACAAAGTGACACTTGCANATGCTGGCTTCATCAAACAATATGGTGATTG  24082
              |||||||||||||||||||||||||||| |||||||||||||||||||||||||||||||
Sbjct  2460   TCTACTTTTCAACAAAGTGACACTTGCAGATGCTGGCTTCATCAAACAATATGGTGATTG  2519

Query  24683  TGATTTTTGTGGTAAGGGCTATCATCTTATGTCCTTCCCTCAGTCAGCACCTCATGGTGT  24742
              |||||||||||| |||||||||||||||||||||||||||||||||||||||||||||||
Sbjct  3120   TGATTTTTGTGGAAAGGGCTATCATCTTATGTCCTTCCCTCAGTCAGCACCTCATGGTGT  3179

Query  25283  CTGTAGTTGTCTCAAGGNCTGTTGTTCTTGTGGATCCTGCTGCAAATTTGATGAAGACGA  25342
              ||||||||||||||||| ||||||||||||||||||||||||||||||||||||||||||
Sbjct  3720   CTGTAGTTGTCTCAAGGGCTGTTGTTCTTGTGGATCCTGCTGCAAATTTGATGAAGACGA  3779
```

In SRR13021137:

Our consensus sequence against the spike gene:

```
Query  1681  CCTTTCCAACAATTTGGCAGAGACATTGCTGACACTACTGATGCTGTCCGTTATCCACAG  1740
             |||||||||||||||||||||||||||||||||||||||||||||||||| |||||||||
Sbjct  1681  CCTTTCCAACAATTTGGCAGAGACATTGCTGACACTACTGATGCTGTCCGTGATCCACAG  1740

Query  1801  GGAACAAATACTTCTAACCAGGTTGCTGTTCTTTATCAGGGTGTTAACTGCACAGAAGTC  1860
             |||||||||||||||||||||||||||||||||||||||| |||||||||||||||||||
Sbjct  1801  GGAACAAATACTTCTAACCAGGTTGCTGTTCTTTATCAGGATGTTAACTGCACAGAAGTC  1860
```

ARTIC's consensus against the spike gene:

```
Query  23243  CCTTTCCAACAATTTGGCAGAGACATTGCTGACACTACTGATGCTGTCCGTNATCCACAG  23302
              ||||||||||||||||||||||||||||||||||||||||||||||||||| ||||||||
Sbjct  1681   CCTTTCCAACAATTTGGCAGAGACATTGCTGACACTACTGATGCTGTCCGTGATCCACAG  1740

Query  23363  GGAACAAATACTTCTAACCAGGTTGCTGTTCTTTATCAGGGTGTTAACTGCACAGAAGTC  23422
              |||||||||||||||||||||||||||||||||||||||| |||||||||||||||||||
Sbjct  1801   GGAACAAATACTTCTAACCAGGTTGCTGTTCTTTATCAGGATGTTAACTGCACAGAAGTC  1860
```

In SRR13021139:

Our consensus sequence against the spike gene:

```
Query  1801   GGAACAAATACTTCTAACCAGGTTGCTGTTCTTTATCAGGGTGTTAACTGCACAGAAGTC  1860
              |||||||||||||||||||||||||||||||||||||||| |||||||||||||||||||
Sbjct  1801   GGAACAAATACTTCTAACCAGGTTGCTGTTCTTTATCAGGATGTTAACTGCACAGAAGTC  1860
```

ARTIC’s consensus against the spike gene:

```
Query  23363  GGAACAAATACTTCTAACCAGGTTGCTGTTCTTTATCAGGNTGTTAACTGCACAGAAGTC  23422
              |||||||||||||||||||||||||||||||||||||||| |||||||||||||||||||
Sbjct  1801   GGAACAAATACTTCTAACCAGGTTGCTGTTCTTTATCAGGATGTTAACTGCACAGAAGTC  1860
```

In SRR13021093:

Our consensus sequence against the spike gene:

```
Query  3601   CAAGAACTTGGAAAGTATGATCAGTATATAAAATGGCCATGGTACATTTGGCTAGGTTTT  3660
              |||||||||||||||||||| |||||||||||||||||||||||||||||||||||||||
Sbjct  3601   CAAGAACTTGGAAAGTATGAGCAGTATATAAAATGGCCATGGTACATTTGGCTAGGTTTT  3660
```

ARTIC’s consensus against the spike gene: 29bp deletion

```
Query  23543  GAGTGTGACA-----------------------------TATCAGACTCAGACTAATTCT  23573
              ||||||||||                             |||||||||||||||||||||
Sbjct  1981   GAGTGTGACATACCCATTGGTGCAGGTATATGCGCTAGTTATCAGACTCAGACTAATTCT  2040

Query  25134  CAAGAACTTGGAAAGTATGATCAGTATATAAAATGGCCATGGTACATTTGGCTAGGTTTT  25193
              |||||||||||||||||||| |||||||||||||||||||||||||||||||||||||||
Sbjct  3601   CAAGAACTTGGAAAGTATGAGCAGTATATAAAATGGCCATGGTACATTTGGCTAGGTTTT  3660
```

Furthermore, based on the publication (1), we identified two accession numbers of the five inconsistent samples, as listed below, and confirmed there were gaps (‘N’) in the sequences.
https://www.ncbi.nlm.nih.gov/sra?linkname=bioproject_sra_all&from_uid=675364

Run	Sample	GISAID virus name	Accession
SRR13021047	nCoV_030	NA	NA
SRR13021061	nCoV_250	hCoV-19/Australia/NSW2250/2020	EPI_ISL_500689
SRR13021093	nCoV_214	hCoV-19/Australia/NSW2214/2020	EPI_ISL_500668
SRR13021137	nCoV_082	hCoV-19/Australia/NSW2082/2020	NA
SRR13021139	nCoV_016	NA	NA

The file SRR13021093.primertrimmed.rg.sorted.bam produced by ARTIC protocol along with the reference genome were used to check the alignment using Tablet (2):

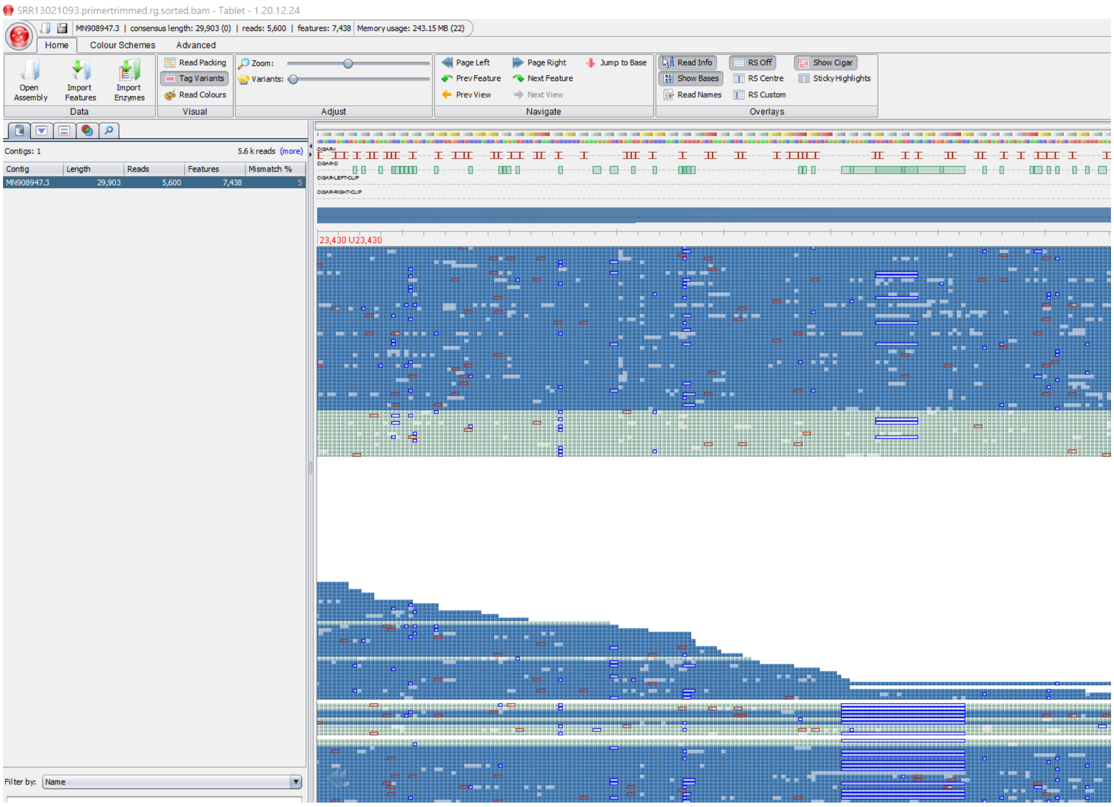

To focus on the region containing the 29-bp delection:

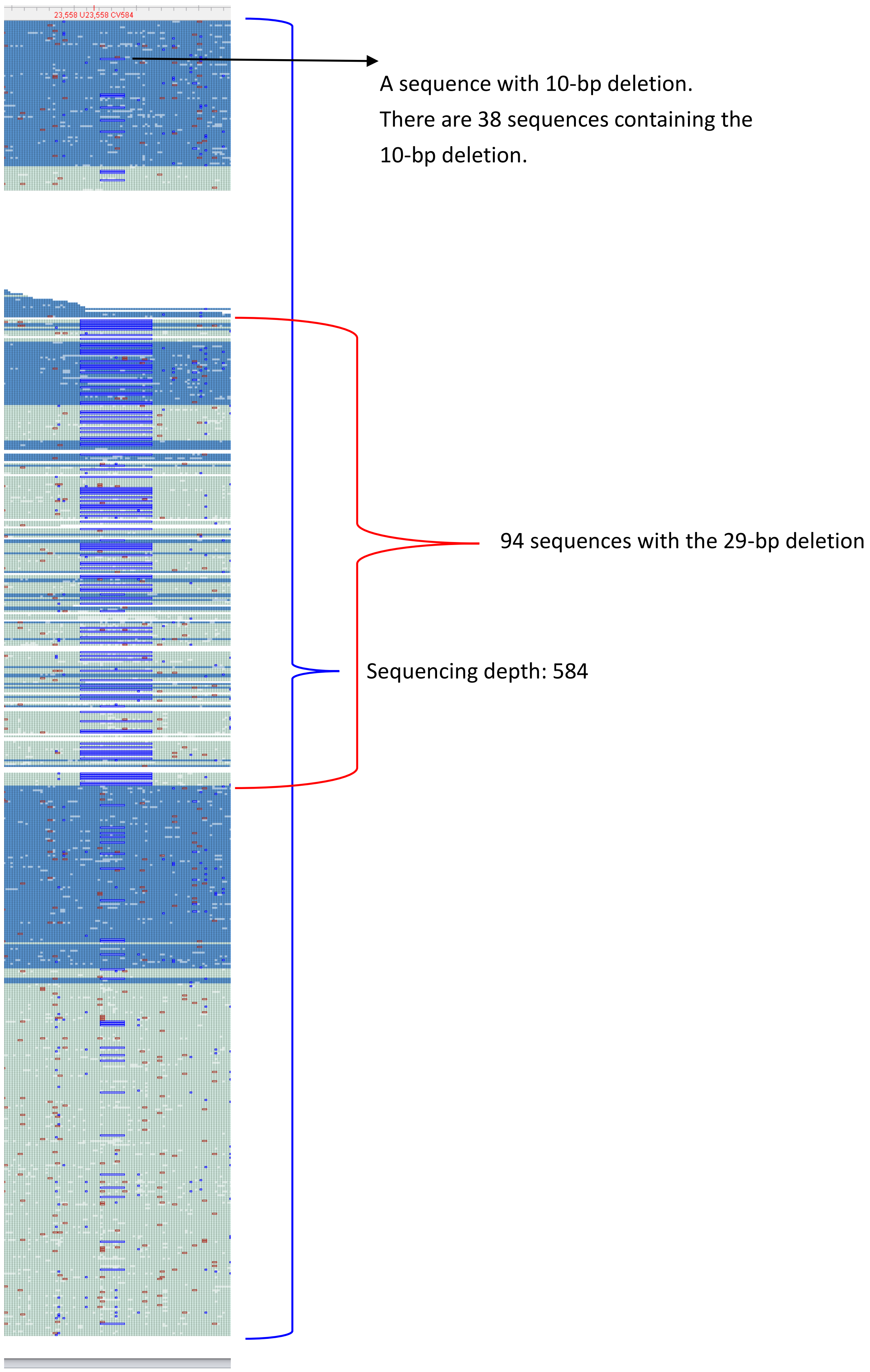
23,558 U23,558 CV584
A sequence with 10-bp deletion.
There are 38 sequences containing the 10-bp deletion.
94 sequences with the 29-bp deletion
Sequencing depth: 584

Similarly, SRR13021093.sorted.bam produced by ARTIC protocol along with the reference genome were used to check the alignment using Interative Genomics Viewer (IGV) (3):

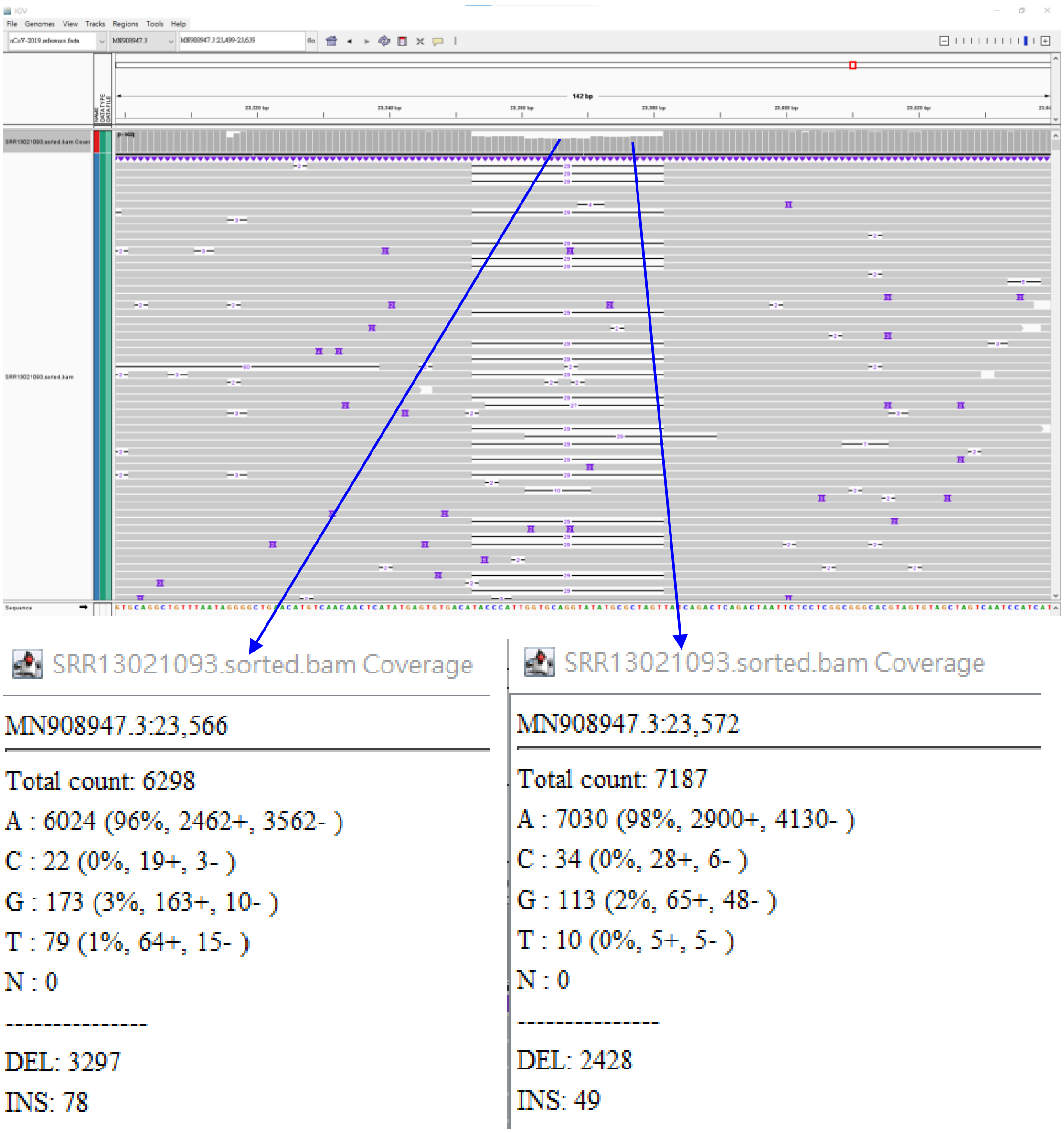

3297/(6298+3297)=34.3%

2428/(7187+2428)=25.3%

We therefore estimated that the sequence proportion containing the 29-bp deletion was less than 30%.

To run covid19S on PRJNA645718

PRJNA645718 (https://www.ncbi.nlm.nih.gov/bioproject/PRJNA645718), library preparation was performed using SARS-CoV-2-Midnight protocol (Oxford Nanopore sequences of 1200 bp amplicon sequences for five SARS-CoV-2 samples) (4).

root@covid19:~/Run# downloadSRA.py -o PRJNA645718 --project PRJNA645718

```
root@covid19:~/Run# downloadSRA.py -o PRJNA645718 --project PRJNA645718
wget 'http://trace.ncbi.nlm.nih.gov/Traces/sra/sra.cgi?save=efetch&rettype=runinfo&db=sra&term=PRJNA645718' -O - > sralist.csv
['OXFORD_NANOPORE']
OXFORD_NANOPORE
          Run          ReleaseDate             LoadDate   spots     bases  ...  Submission  dbgap_study_accession  Consent                          RunHash                         ReadHash
0  SRR12209729  2020-07-13 12:02:15  2020-07-13 11:58:53  114879  58446889  ...  SRA1098098                    NaN   public  94D766BE3DA824466D6D3105C9D416CF  7F419F3C494C7B222ADFAB84A2014704
1  SRR12209728  2020-07-13 12:02:15  2020-07-13 11:58:44   58899  26918002  ...  SRA1098098                    NaN   public  877E9230B2C290D675A46A1A3DDDCEF4  10E36658F3BCA50152D402461877AF39
2  SRR12209727  2020-07-13 12:02:15  2020-07-13 11:58:49   56842  26118431  ...  SRA1098098                    NaN   public  4005EDCC86DC05168D363E0EDC0D2F68  D7FB53F83CC5BC2722843E6C5B90AC89
3  SRR12209726  2020-07-13 12:02:15  2020-07-13 11:58:47   53001  25871724  ...  SRA1098098                    NaN   public  8E3B7ACB46FFFAFC9CDBE26584BF35D4  9B37FE32C1DBF2B9E6E6B51A4EB4772B
4  SRR12209725  2020-07-13 12:02:15  2020-07-13 11:58:50   45168  22086531  ...  SRA1098098                    NaN   public  9E65D4CA7954C592597F2C37C55D59C6  684616AB3F5E1CDD0DD332DC23D2344E

[5 rows x 47 columns]
          Run          ReleaseDate             LoadDate   spots     bases  ...  Submission  dbgap_study_accession  Consent                          RunHash                         ReadHash
0  SRR12209729  2020-07-13 12:02:15  2020-07-13 11:58:53  114879  58446889  ...  SRA1098098                    NaN   public  94D766BE3DA824466D6D3105C9D416CF  7F419F3C494C7B222ADFAB84A2014704
1  SRR12209728  2020-07-13 12:02:15  2020-07-13 11:58:44   58899  26918002  ...  SRA1098098                    NaN   public  877E9230B2C290D675A46A1A3DDDCEF4  10E36658F3BCA50152D402461877AF39
2  SRR12209727  2020-07-13 12:02:15  2020-07-13 11:58:49   56842  26118431  ...  SRA1098098                    NaN   public  4005EDCC86DC05168D363E0EDC0D2F68  D7FB53F83CC5BC2722843E6C5B90AC89
3  SRR12209726  2020-07-13 12:02:15  2020-07-13 11:58:47   53001  25871724  ...  SRA1098098                    NaN   public  8E3B7ACB46FFFAFC9CDBE26584BF35D4  9B37FE32C1DBF2B9E6E6B51A4EB4772B
4  SRR12209725  2020-07-13 12:02:15  2020-07-13 11:58:50   45168  22086531  ...  SRA1098098                    NaN   public  9E65D4CA7954C592597F2C37C55D59C6  684616AB3F5E1CDD0DD332DC23D2344E

[5 rows x 47 columns]
['SRR12209729' 'SRR12209728' 'SRR12209727' 'SRR12209726' 'SRR12209725']
fastq-dump SRR12209729
fastq-dump SRR12209728
fastq-dump SRR12209727
fastq-dump SRR12209726
fastq-dump SRR12209725
```

A folder named PRJNA645718 was produced to contain the 5 corresponding FASTQ files:

```
root@covid19:~/Run/PRJNA645718# ll
total 335088
drwxr-xr-x 2 root root      4096 May 11 15:19 ./
drwxr-xr-x 4 root root      4096 May 11 15:15 ../
-rw-r--r-- 1 root root  47478886 May 11 15:19 SRR12209725.fastq
-rw-r--r-- 1 root root  55631076 May 11 15:19 SRR12209726.fastq
-rw-r--r-- 1 root root  56406196 May 11 15:19 SRR12209727.fastq
-rw-r--r-- 1 root root  58159154 May 11 15:19 SRR12209728.fastq
-rw-r--r-- 1 root root 125430182 May 11 15:20 SRR12209729.fastq
-rw-r--r-- 1 root root      2982 May 11 15:18 sralist.csv
```

root@covid19:~/Run# runconsensus.py -i PRJNA645718/ -o PRJNA645718/01_consensus

```
root@covid19:~/Run# runconsensus.py -i PRJNA645718/ -o PRJNA645718/01_consensus
root@covid19:~/Run# cd PRJNA645718/
root@covid19:~/Run/PRJNA645718# ll
total 335092
drwxr-xr-x 3 root root      4096 May 11 15:31 ./
drwxr-xr-x 4 root root      4096 May 11 15:15 ../
drwxr-xr-x 7 root root      4096 May 11 15:35 01_consensus/
-rw-r--r-- 1 root root  47478886 May 11 15:19 SRR12209725.fastq
-rw-r--r-- 1 root root  55631076 May 11 15:19 SRR12209726.fastq
-rw-r--r-- 1 root root  56406196 May 11 15:19 SRR12209727.fastq
-rw-r--r-- 1 root root  58159154 May 11 15:19 SRR12209728.fastq
-rw-r--r-- 1 root root 125430182 May 11 15:20 SRR12209729.fastq
-rw-r--r-- 1 root root      2982 May 11 15:18 sralist.csv
root@covid19:~/Run/PRJNA645718# cd 01_consensus/
root@covid19:~/Run/PRJNA645718/01_consensus# ll
total 828
drwxr-xr-x 7 root root   4096 May 11 15:35 ./
drwxr-xr-x 3 root root   4096 May 11 15:31 ../
drwxr-xr-x 2 root root   4096 May 11 15:31 SRR12209725/
-rw-r--r-- 1 root root   3856 May 11 15:32 SRR12209725_final.fa
-rw-r--r-- 1 root root      0 May 11 15:31 SRR12209725_md.txt
-rw-r--r-- 1 root root   3856 May 11 15:31 SRR12209725_ref_1.fa
-rw-r--r-- 1 root root     43 May 11 15:31 SRR12209725_ref_1.fa.fai
-rw-r--r-- 1 root root 144573 May 11 15:31 SRR12209725_ref_1.fa.mmi
drwxr-xr-x 2 root root   4096 May 11 15:32 SRR12209726/
-rw-r--r-- 1 root root   3856 May 11 15:33 SRR12209726_final.fa
-rw-r--r-- 1 root root      0 May 11 15:32 SRR12209726_md.txt
-rw-r--r-- 1 root root   3856 May 11 15:32 SRR12209726_ref_1.fa
-rw-r--r-- 1 root root     43 May 11 15:32 SRR12209726_ref_1.fa.fai
-rw-r--r-- 1 root root 144589 May 11 15:32 SRR12209726_ref_1.fa.mmi
drwxr-xr-x 2 root root   4096 May 11 15:33 SRR12209727/
-rw-r--r-- 1 root root   3856 May 11 15:34 SRR12209727_final.fa
-rw-r--r-- 1 root root      0 May 11 15:33 SRR12209727_md.txt
-rw-r--r-- 1 root root   3856 May 11 15:33 SRR12209727_ref_1.fa
-rw-r--r-- 1 root root     43 May 11 15:33 SRR12209727_ref_1.fa.fai
-rw-r--r-- 1 root root 144605 May 11 15:33 SRR12209727_ref_1.fa.mmi
drwxr-xr-x 2 root root   4096 May 11 15:34 SRR12209728/
-rw-r--r-- 1 root root   3856 May 11 15:34 SRR12209728_final.fa
-rw-r--r-- 1 root root      0 May 11 15:34 SRR12209728_md.txt
-rw-r--r-- 1 root root   3856 May 11 15:34 SRR12209728_ref_1.fa
-rw-r--r-- 1 root root     43 May 11 15:34 SRR12209728_ref_1.fa.fai
-rw-r--r-- 1 root root 144573 May 11 15:34 SRR12209728_ref_1.fa.mmi
drwxr-xr-x 2 root root   4096 May 11 15:35 SRR12209729/
-rw-r--r-- 1 root root   3856 May 11 15:35 SRR12209729_final.fa
-rw-r--r-- 1 root root      0 May 11 15:35 SRR12209729_md.txt
-rw-r--r-- 1 root root   3856 May 11 15:35 SRR12209729_ref_1.fa
-rw-r--r-- 1 root root     43 May 11 15:35 SRR12209729_ref_1.fa.fai
-rw-r--r-- 1 root root 144589 May 11 15:35 SRR12209729_ref_1.fa.mmi
-rw-r--r-- 1 root root  19180 May 11 15:35 consensus.fasta
root@covid19:~/Run/PRJNA645718/01_consensus#
```

Consensus sequences for each samples were produced (*_final.fa). These files were concatenated to form a consensus.fasta if the consensus sequence was single and long (>3000 bp).

A consensus.fasta was produce to contain the consensus sequences of samples. This file was able to be uploaded to Nextclade (https://clades.nextstrain.org/) for mutation calling. The result was exported and renamed to a TSV file (PRJNA645718.tsv).

```
root@covid19:~/Run/PRJNA645718/01_consensus# mv consensus.fasta PRJNA645718_consensus.fasta
```

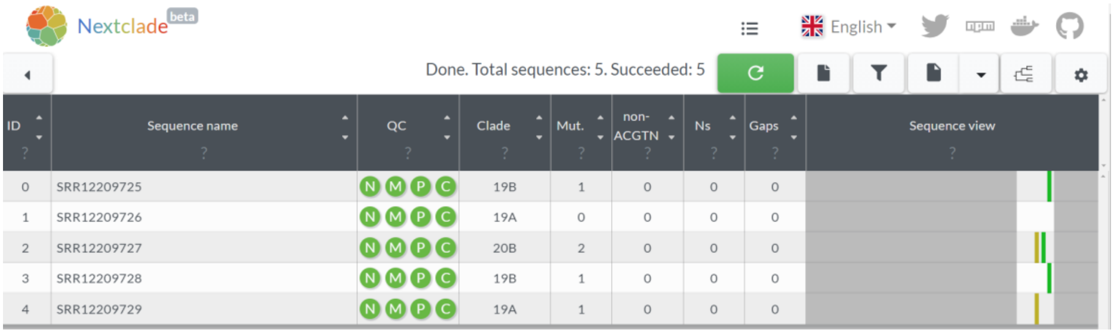

ID	Sequence name	QC	Clade	Mut.	non-ACGTN	Ns	Gaps	Sequence view
0	SRR12209725	N M P C	19B	1	0	0	0	
1	SRR12209726	N M P C	19A	0	0	0	0	
2	SRR12209727	N M P C	20B	2	0	0	0	
3	SRR12209728	N M P C	19B	1	0	0	0	
4	SRR12209729	N M P C	19A	1	0	0	0	

seqName	clade	aaSubstitutions (in spike protein)
SRR12209725	19B	
SRR12209726	19A	
SRR12209727	20B	S:D614G,S:A846V
SRR12209728	19B	
SRR12209729	19A	S:D614G

To produce spike protein variants:

root@covid19:~/Run# getvar.py -i PRJNA645718/01_consensus/ -o PRJNA645718/02_output -q PRJNA645718/

```
root@covid19:~/Run# getvar.py -i PRJNA645718/01_consensus/ -o PRJNA645718/02_output -q PRJNA645718/
root@covid19:~/Run# cd PRJNA645718/02_output/
root@covid19:~/Run/PRJNA645718/02_output# ll
total 60
drwxr-xr-x 2 root root  4096 May 11 15:55 ./
drwxr-xr-x 4 root root  4096 May 11 15:55 ../
-rw-r--r-- 1 root root 25591 May 11 15:55 Result.csv
-rw-r--r-- 1 root root 19180 May 11 15:55 output.fasta
-rw-r--r-- 1 root root    81 May 11 15:55 output.txt
```

```
root@covid19:~/Run/PRJNA645718/02_output# cat output.txt
SRR12209725
SRR12209726
SRR12209727     D614G;A846V
SRR12209728
SRR12209729     D614G
```

root@covid19:~/Run/PRJNA645718/02_output# mv Result.csv PRJNA645718_Result.csv

A file named Result.csv was produced to show spike variants along with nucleotide and amino acid sequences.

	A	B	C	D
1	Name	Variants	NT	AA
2	SRR12209725		ATGTTTGTTTTTCTTGTTTT	MFVFLVLLPLVSSQCVNLTTRTQLPP
3	SRR12209726		ATGTTTGTTTTTCTTGTTTT	MFVFLVLLPLVSSQCVNLTTRTQLPP
4	SRR12209727	D614G;A846V	ATGTTTGTTTTTCTTGTTTT	MFVFLVLLPLVSSQCVNLTTRTQLPP
5	SRR12209728		ATGTTTGTTTTTCTTGTTTT	MFVFLVLLPLVSSQCVNLTTRTQLPP
6	SRR12209729	D614G	ATGTTTGTTTTTCTTGTTTT	MFVFLVLLPLVSSQCVNLTTRTQLPP

```
root@covid19:~/Run# seqkit stats PRJNA645718/*.fastq
file                            format  type  num_seqs     sum_len  min_len  avg_len  max_len
PRJNA645718/SRR12209725.fastq   FASTQ   DNA     45,168  22,086,531       77      489    2,688
PRJNA645718/SRR12209726.fastq   FASTQ   DNA     53,001  25,871,724       62    488.1    5,122
PRJNA645718/SRR12209727.fastq   FASTQ   DNA     56,842  26,118,431       77    459.5   14,541
PRJNA645718/SRR12209728.fastq   FASTQ   DNA     58,899  26,918,002       76      457   28,419
PRJNA645718/SRR12209729.fastq   FASTQ   DNA    114,879  58,446,889       70    508.8    6,124
```

root@covid19:~/Run# runtrimming.py -i PRJNA645718/ -o PRJNA645718_trimmed

```
root@covid19:~/Run# runtrimming.py -i PRJNA645718/ -o PRJNA645718_trimmed
cat /root/Run/PRJNA645718/SRR12209727.fastq | seqkit subseq -r 100:-100 > /root/Run/PRJNA645718_trimmed/SRR12209727.fastq
cat /root/Run/PRJNA645718/SRR12209726.fastq | seqkit subseq -r 100:-100 > /root/Run/PRJNA645718_trimmed/SRR12209726.fastq
cat /root/Run/PRJNA645718/SRR12209729.fastq | seqkit subseq -r 100:-100 > /root/Run/PRJNA645718_trimmed/SRR12209729.fastq
cat /root/Run/PRJNA645718/SRR12209728.fastq | seqkit subseq -r 100:-100 > /root/Run/PRJNA645718_trimmed/SRR12209728.fastq
cat /root/Run/PRJNA645718/SRR12209725.fastq | seqkit subseq -r 100:-100 > /root/Run/PRJNA645718_trimmed/SRR12209725.fastq
```

```
root@covid19:~/Run# seqkit stats PRJNA645718_trimmed/*.fastq
file                                    format  type  num_seqs     sum_len  min_len  avg_len  max_len
PRJNA645718_trimmed/SRR12209725.fastq   FASTQ   DNA     45,168  13,232,625        0      293    2,490
PRJNA645718_trimmed/SRR12209726.fastq   FASTQ   DNA     53,001  15,465,227        0    291.8    4,924
PRJNA645718_trimmed/SRR12209727.fastq   FASTQ   DNA     56,842  14,959,973        0    263.2   14,343
PRJNA645718_trimmed/SRR12209728.fastq   FASTQ   DNA     58,899  15,389,777        0    261.3   28,221
PRJNA645718_trimmed/SRR12209729.fastq   FASTQ   DNA    114,879  35,815,272        0    311.8    5,926
```

root@covid19:~/Run# runconsensus.py -i PRJNA645718_trimmed/ -o PRJNA645718_trimmed/01_consensus

root@covid19:~/Run# getvar.py -i PRJNA645718_trimmed/01_consensus/ -o PRJNA645718_trimmed/02_output -q PRJNA645718_trimmed/

root@covid19:~/Run# cat PRJNA645718_trimmed/02_output/output.txt

```
root@covid19:~/Run# cat PRJNA645718_trimmed/02_output/output.txt
SRR12209725
SRR12209726
SRR12209727     D614G;A846V
SRR12209728
SRR12209729     D614G
```

To run covid19S on PRJNA694014

PRJNA694014 (https://www.ncbi.nlm.nih.gov/bioproject/?term=PRJNA694014), library preparation was performed using the ARTIC V3 tiling method (5).

root@covid19:~/Run# downloadSRA.py -o PRJNA694014 --project PRJNA694014 --nanopore T

```
root@covid19:~/Run# downloadSRA.py -o PRJNA694014 --project PRJNA694014 --nanopore T
wget 'http://trace.ncbi.nlm.nih.gov/Traces/sra/sra.cgi?save=efetch&rettype=runinfo&db=sra&term=PRJNA694014' -O - > sralist.csv
['ILLUMINA' 'OXFORD_NANOPORE']
OXFORD_NANOPORE
            Run          ReleaseDate             LoadDate   spots      bases  ...  Submission  dbgap_study_accession  Consent
0    SRR13620361  2021-02-03 14:07:10  2021-02-03 13:58:57  279204  106511514  ...  SRA1191127                    NaN   public  C97
1    SRR13620350  2021-02-03 14:07:12  2021-02-03 13:59:27  267260   81294119  ...  SRA1191127                    NaN   public  01E
2    SRR13620339  2021-02-03 14:07:15  2021-02-03 13:58:50  333583  115590863  ...  SRA1191127                    NaN   public  4E1
3    SRR13620328  2021-02-03 14:07:18  2021-02-03 13:58:30  320060  118283749  ...  SRA1191127                    NaN   public  FF6
4    SRR13620416  2021-02-03 16:46:44  2021-02-03 14:00:16  323054  122750343  ...  SRA1191127                    NaN   public  0AA
..           ...                  ...                  ...     ...        ...  ...         ...                    ...      ...
395  SRR13620231  2021-02-03 14:00:19  2021-02-03 13:56:25  422203  134423312  ...  SRA1191127                    NaN   public  13E
396  SRR13620230  2021-02-03 14:00:20  2021-02-03 13:56:23  331225  130012719  ...  SRA1191127                    NaN   public  C4E
397  SRR13620229  2021-02-03 14:00:20  2021-02-03 13:56:17  407746  134580000  ...  SRA1191127                    NaN   public  BC4
398  SRR13620228  2021-02-03 13:57:21  2021-02-03 13:56:12  221713   69502241  ...  SRA1191127                    NaN   public  2BD
399  SRR13620226  2021-02-03 14:00:20  2021-02-03 13:56:20  444374  139972580  ...  SRA1191127                    NaN   public  C5F

[400 rows x 47 columns]
            Run          ReleaseDate             LoadDate   spots      bases  ...  Submission  dbgap_study_accession  Consent
41   SRR13620425  2021-02-03 16:46:43  2021-02-03 14:00:12  325345  159348807  ...  SRA1191127                    NaN   public  347
42   SRR13620424  2021-02-03 16:46:43  2021-02-03 14:00:37  341162  171269720  ...  SRA1191127                    NaN   public  F20
43   SRR13620423  2021-02-03 16:46:43  2021-02-03 14:00:42  288641   99564489  ...  SRA1191127                    NaN   public  AE0
44   SRR13620422  2021-02-03 16:46:43  2021-02-03 14:00:51  394772  199431794  ...  SRA1191127                    NaN   public  F59
45   SRR13620421  2021-02-03 14:07:04  2021-02-03 13:59:49  141169   39879092  ...  SRA1191127                    NaN   public  763
..           ...                  ...                  ...     ...        ...  ...         ...                    ...      ...
195  SRR13620431  2021-02-03 16:46:43  2021-02-03 14:00:10   72463   16745473  ...  SRA1191127                    NaN   public  6A7
196  SRR13620430  2021-02-03 16:46:43  2021-02-03 14:03:06  434427  222894421  ...  SRA1191127                    NaN   public  1B1
197  SRR13620429  2021-02-03 16:46:43  2021-02-03 14:00:08   51006   11506630  ...  SRA1191127                    NaN   public  1F1
198  SRR13620428  2021-02-03 16:46:43  2021-02-03 14:00:27   98366   33728636  ...  SRA1191127                    NaN   public  516
199  SRR13620426  2021-02-03 16:46:43  2021-02-03 14:00:33  341055  169538061  ...  SRA1191127                    NaN   public  9D9

[64 rows x 47 columns]
['SRR13620425' 'SRR13620424' 'SRR13620423' 'SRR13620422' 'SRR13620421'
 'SRR13620420' 'SRR13620419' 'SRR13620418' 'SRR13620417' 'SRR13620415'
 'SRR13620414' 'SRR13620413' 'SRR13620412' 'SRR13620411' 'SRR13620410'
 'SRR13620409' 'SRR13620408' 'SRR13620407' 'SRR13620406' 'SRR13620404'
 'SRR13620403' 'SRR13620402' 'SRR13620401' 'SRR13620400' 'SRR13620399'
 'SRR13620398' 'SRR13620397' 'SRR13620396' 'SRR13620395' 'SRR13620393'
 'SRR13620392' 'SRR13620391' 'SRR13620390' 'SRR13620389' 'SRR13620388'
 'SRR13620387' 'SRR13620386' 'SRR13620385' 'SRR13620384' 'SRR13620382'
 'SRR13620381' 'SRR13620380' 'SRR13620379' 'SRR13620378' 'SRR13620377'
 'SRR13620376' 'SRR13620375' 'SRR13620374' 'SRR13620373' 'SRR13620371'
 'SRR13620370' 'SRR13620369' 'SRR13620368' 'SRR13620367' 'SRR13620366'
 'SRR13620365' 'SRR13620364' 'SRR13620363' 'SRR13620362' 'SRR13620431'
 'SRR13620430' 'SRR13620429' 'SRR13620428' 'SRR13620426']
fastq-dump SRR13620425
fastq-dump SRR13620424
```

```
root@covid19:~/Run# cd PRJNA694014/
root@covid19:~/Run/PRJNA694014# ll *.fastq | wc -l
64
root@covid19:~/Run/PRJNA694014# ll *.fastq | head
-rw-r--r-- 1 root root   92879494 May 11 16:10 SRR13620362.fastq
-rw-r--r-- 1 root root  212316312 May 11 16:10 SRR13620363.fastq
-rw-r--r-- 1 root root 1725259338 May 11 16:34 SRR13620364.fastq
-rw-r--r-- 1 root root  269475236 May 11 16:08 SRR13620365.fastq
-rw-r--r-- 1 root root 2397126262 May 11 16:41 SRR13620366.fastq
-rw-r--r-- 1 root root  598674238 May 11 16:15 SRR13620367.fastq
-rw-r--r-- 1 root root  785412394 May 11 16:17 SRR13620368.fastq
-rw-r--r-- 1 root root 1439473640 May 11 16:29 SRR13620369.fastq
-rw-r--r-- 1 root root  784187366 May 11 16:20 SRR13620370.fastq
-rw-r--r-- 1 root root  304940774 May 11 16:10 SRR13620371.fastq
```

To trim out adapter and barcode sequences:

```
usage: runtrimming.py [-h] [-i I] [-o O] [-t T] [-l L]

optional arguments:
  -h, --help  show this help message and exit
  -i I        an input folder
  -o O        an output folder
  -t T        threads (default=100)
  -l L        trimming length (default=100 bp)
```

root@covid19:~/Run# runtrimming.py -i PRJNA694014/ -o PRJNA694014_trimmed

root@covid19:~/Run# runconsensus.py -i PRJNA694014_trimmed/ -o PRJNA694014_trimmed/01_consensus

```
root@covid19:~/Run# runconsensus.py -i PRJNA694014_trimmed/ -o PRJNA694014_trimmed/01_consensus
SRR13620365 was not included in the consensus.fasta
SRR13620373 was not included in the consensus.fasta
SRR13620376 was not included in the consensus.fasta
SRR13620377 was not included in the consensus.fasta
SRR13620378 was not included in the consensus.fasta
SRR13620390 was not included in the consensus.fasta
SRR13620391 was not included in the consensus.fasta
SRR13620396 was not included in the consensus.fasta
SRR13620397 was not included in the consensus.fasta
SRR13620399 was not included in the consensus.fasta
SRR13620401 was not included in the consensus.fasta
SRR13620402 was not included in the consensus.fasta
SRR13620403 was not included in the consensus.fasta
SRR13620404 was not included in the consensus.fasta
SRR13620407 was not included in the consensus.fasta
SRR13620410 was not included in the consensus.fasta
SRR13620411 was not included in the consensus.fasta
SRR13620412 was not included in the consensus.fasta
SRR13620418 was not included in the consensus.fasta
SRR13620421 was not included in the consensus.fasta
SRR13620422 was not included in the consensus.fasta
SRR13620423 was not included in the consensus.fasta
SRR13620425 was not included in the consensus.fasta
SRR13620426 was not included in the consensus.fasta
SRR13620428 was not included in the consensus.fasta
SRR13620429 was not included in the consensus.fasta
SRR13620431 was not included in the consensus.fasta
```

```
root@covid19:~/Run# cd PRJNA694014_trimmed/01_consensus/
root@covid19:~/Run/PRJNA694014_trimmed/01_consensus# grep '>' consensus.fasta | wc -l
37
```

root@covid19:~/Run/PRJNA694014_trimmed/01_consensus# cd ..
root@covid19:~/Run/PRJNA694014_trimmed# cd ..
root@covid19:~/Run# mv PRJNA694014_trimmed/01_consensus/consensus.fasta /PRJNA694014_consensus.fasta

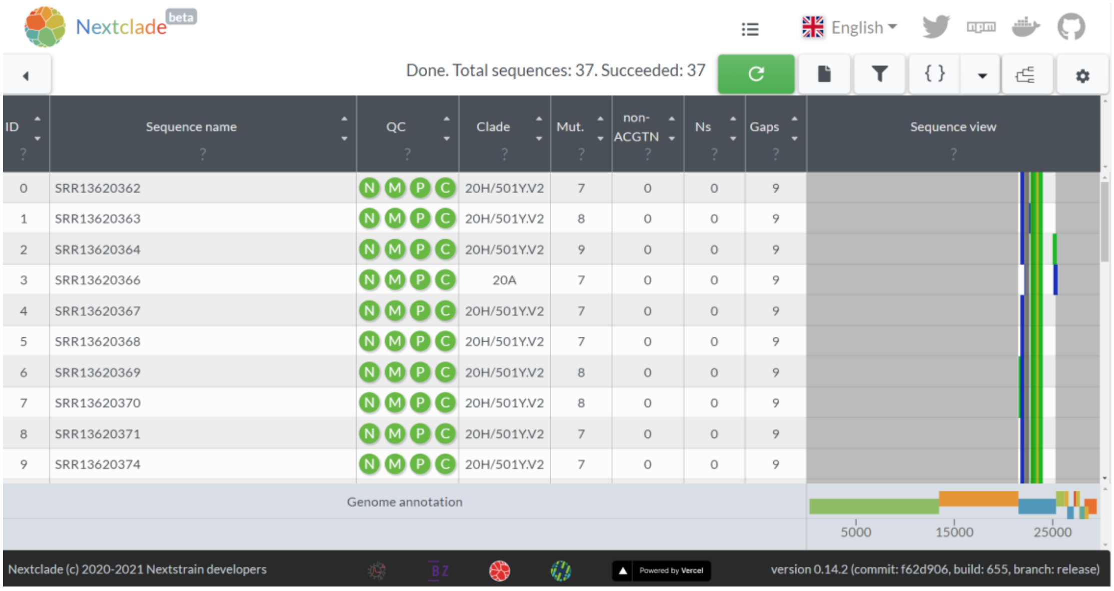

ID	Sequence name	QC	Clade	Mut.	non-ACGTN	Ns	Gaps
0	SRR13620362	N M P C	20H/501Y.V2	7	0	0	9
1	SRR13620363	N M P C	20H/501Y.V2	8	0	0	9
2	SRR13620364	N M P C	20H/501Y.V2	9	0	0	9
3	SRR13620366	N M P C	20A	7	0	0	9
4	SRR13620367	N M P C	20H/501Y.V2	7	0	0	9
5	SRR13620368	N M P C	20H/501Y.V2	7	0	0	9
6	SRR13620369	N M P C	20H/501Y.V2	8	0	0	9
7	SRR13620370	N M P C	20H/501Y.V2	8	0	0	9
8	SRR13620371	N M P C	20H/501Y.V2	7	0	0	9
9	SRR13620374	N M P C	20H/501Y.V2	7	0	0	9

root@covid19:~/Run# getvar.py -i PRJNA694014_trimmed/01_consensus/ -o

PRJNA694014_trimmed/02_output -q PRJNA694014_trimmed/

```
root@covid19:~/Run# getvar.py -i PRJNA694014_trimmed/01_consensus/ -o PRJNA694014_trimmed/02_output -q PRJNA694014_trimmed/
SRR13620375_final.fa can not be corrected, try to checck sequencing depth...
SRR13620385_final.fa can not be corrected, try to checck sequencing depth...
SRR13620392_final.fa can not be corrected, try to checck sequencing depth...
SRR13620408_final.fa can not be corrected, try to checck sequencing depth...
SRR13620413_final.fa can not be corrected, try to checck sequencing depth...
  gap in subject:GAAAAAAGGAA==>GAAAAAGGAA
SRR13620414_final.fa can not be corrected, try to checck sequencing depth...
```

root@covid19:~/Run# grep 'No' PRJNA694014_trimmed/02_output/output.txt | wc -l

27

root@covid19:~/Run# grep 'Segment' PRJNA694014_trimmed/02_output/output.txt | wc -l

15

root@covid19:~/Run# mv PRJNA694014_trimmed/02_output/Result.csv PRJNA694014_Result.csv

```
root@covid19:~/Run/PRJNA694014_trimmed/01_consensus_rerun# ll *_ref_5*.fa
-rw-r--r-- 1 root root 3770 May 12 09:22 SRR13620365_ref_5.fa
-rw-r--r-- 1 root root 3855 May 12 09:34 SRR13620373_ref_5.fa
-rw-r--r-- 1 root root 3829 May 12 09:38 SRR13620376_ref_5.fa
-rw-r--r-- 1 root root 3853 May 12 09:41 SRR13620377_ref_5.fa
-rw-r--r-- 1 root root 3856 May 12 09:44 SRR13620378_ref_5.fa
-rw-r--r-- 1 root root 3843 May 12 09:59 SRR13620390_ref_5.fa
-rw-r--r-- 1 root root 3855 May 12 10:01 SRR13620391_ref_5.fa
-rw-r--r-- 1 root root 3776 May 12 10:09 SRR13620396_ref_5.fa
-rw-r--r-- 1 root root 3724 May 12 10:12 SRR13620397_ref_5.fa
-rw-r--r-- 1 root root 3716 May 12 10:16 SRR13620399_ref_5.fa
-rw-r--r-- 1 root root 3853 May 12 10:20 SRR13620401_ref_5.fa
-rw-r--r-- 1 root root 3752 May 12 10:24 SRR13620402_ref_5.fa
-rw-r--r-- 1 root root 3741 May 12 10:27 SRR13620403_ref_5.fa
-rw-r--r-- 1 root root 3869 May 12 10:29 SRR13620404_ref_5.fa
-rw-r--r-- 1 root root 3853 May 12 10:35 SRR13620407_ref_5.fa
-rw-r--r-- 1 root root 3710 May 12 10:40 SRR13620410_ref_5.fa
-rw-r--r-- 1 root root 3873 May 12 10:42 SRR13620411_ref_5.fa
-rw-r--r-- 1 root root 3839 May 12 10:45 SRR13620412_ref_5.fa
-rw-r--r-- 1 root root 3826 May 12 10:54 SRR13620418_ref_5.fa
-rw-r--r-- 1 root root 3856 May 12 11:00 SRR13620421_ref_5.fa
-rw-r--r-- 1 root root 3854 May 12 11:03 SRR13620422_ref_5.fa
-rw-r--r-- 1 root root 4103 May 12 11:05 SRR13620423_ref_5.fa
-rw-r--r-- 1 root root 3854 May 12 11:11 SRR13620425_ref_5.fa
-rw-r--r-- 1 root root 3853 May 12 11:14 SRR13620426_ref_5.fa
-rw-r--r-- 1 root root 3869 May 12 11:16 SRR13620428_ref_5.fa
root@covid19:~/Run/PRJNA694014_trimmed/01_consensus_rerun# ll *_ref_5*.fa |wc -l
25
```

To run consensus without trimming:

root@covid19:~/Run# runconsensus.py -i PRJNA694014 -o PRJNA694014/01_consensus

```
root@covid19:~/Run# runconsensus.py -i PRJNA694014 -o PRJNA694014/01_consensus
SRR13620365 was not included in the consensus.fasta
SRR13620371 was not included in the consensus.fasta
SRR13620374 was not included in the consensus.fasta
SRR13620376 was not included in the consensus.fasta
SRR13620378 was not included in the consensus.fasta
SRR13620379 was not included in the consensus.fasta
SRR13620382 was not included in the consensus.fasta
SRR13620390 was not included in the consensus.fasta
SRR13620391 was not included in the consensus.fasta
SRR13620393 was not included in the consensus.fasta
SRR13620395 was not included in the consensus.fasta
SRR13620396 was not included in the consensus.fasta
SRR13620397 was not included in the consensus.fasta
SRR13620399 was not included in the consensus.fasta
SRR13620401 was not included in the consensus.fasta
SRR13620403 was not included in the consensus.fasta
SRR13620404 was not included in the consensus.fasta
SRR13620406 was not included in the consensus.fasta
SRR13620410 was not included in the consensus.fasta
SRR13620411 was not included in the consensus.fasta
SRR13620412 was not included in the consensus.fasta
SRR13620418 was not included in the consensus.fasta
SRR13620421 was not included in the consensus.fasta
SRR13620428 was not included in the consensus.fasta
SRR13620429 was not included in the consensus.fasta
SRR13620430 was not included in the consensus.fasta
SRR13620431 was not included in the consensus.fasta
root@covid19:~/Run# grep '>' PRJNA694014/01_consensus/consensus.fasta | wc -l
37
```

The above consensus sequences are listed in Supplementary Table S2 (PRJNA694014_consensus).

root@covid19:~/Run# getvar.py -i PRJNA694014/01_consensus/ -o PRJNA694014/02_output -q PRJNA694014

```
root@covid19:~/Run# getvar.py -i PRJNA694014/01_consensus/ -o PRJNA694014/02_output -q PRJNA694014
SRR13620392_final.fa can not be corrected, try to checck sequencing depth...
```

root@covid19:~/Run# grep 'seg' PRJNA694014/02_output/output.txt | wc -l

9

root@covid19:~/Run# grep 'multi' PRJNA694014/02_output/output.txt | wc -l

27

The consensus sequences were uploaded to Nextclade and the exported results are shown in Supplementary Table S2 (PRJNA694014_Nextclade). Besides, the Result.tsv in 02_output is shown in Supplementary Table S2 (PRJNA694014_Result).

Please note that there were differences between the consensus.fasta with trimming and the consensus.fasta without trimming (Supplementary Table S1, PRJNA694014's Our bioinformatic protocol). We therefore combined these to sequences for uploading to Nextclade. (Spike consensus+Nextclade)

```
root@covid19:~/Run# addseq.py PRJNA694014/01_consensus/consensus.fasta
PRJNA694014_trimmed/01_consensus/consensus.fasta
PRJNA694014_all_consensus.fasta
root@covid19:~/Run# grep '>' PRJNA694014_all_consensus.fasta | wc -l
45
```

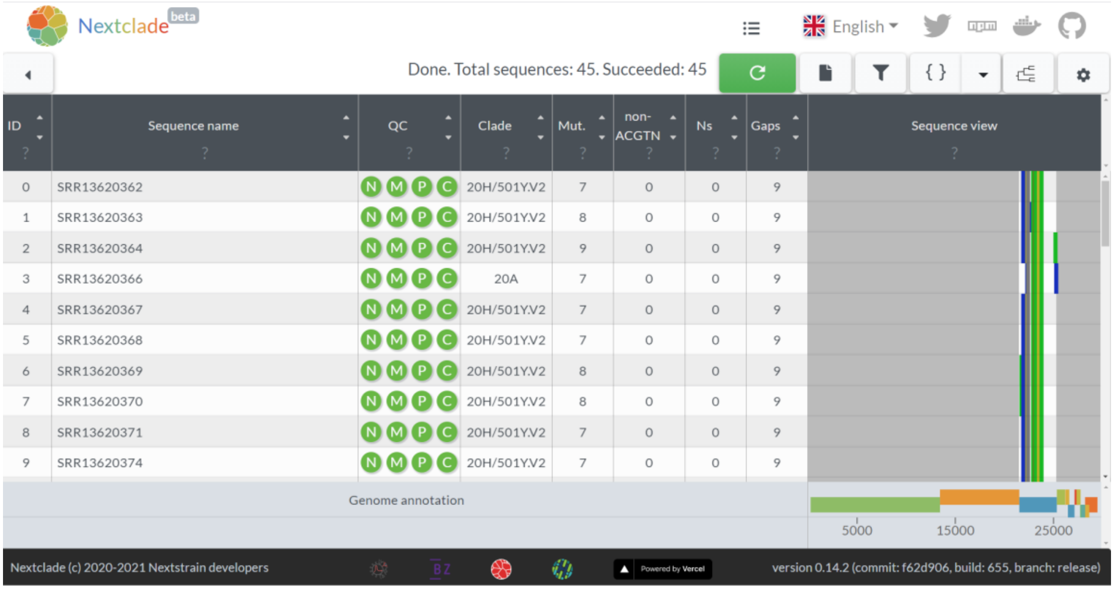

Furthermore, ARTIC protocol was used to run this project data. According to the bioinformatics protocols (https://artic.network/ncov-2019/ncov2019-bioinformatics-sop.html, https://github.com/artic-network/artic-ncov2019), the consensus sequences of SARS-CoV-2 genomes are shown in Supplementary Table S2 (PRJNA694014_ARTIC_consensus). The sequences were uploaded to Next clade for clade assignment and variant calling (PRJNA694014_ARTIC_Nextclade). The spike protein variants of ARTIC results combined with variant calling of Nextclade are shown in Supplementary Table S1 (PRJNA694014’s ARTIC+Nextclade). Please note that the deletions of three amino acids at positions 242 to 244 (6) were miscalled at sites of 243 to 245 for many samples by Nextclade.

To download nanopore sequences of SARS-CoV-2 from SRA

As shown in NCBI SARS-CoV-2 Resources (https://www.ncbi.nlm.nih.gov/sars-cov-2/), we accessed the SRA runs on Feb 18, 2022. By selecting Platform to Oxford Nanopore, 276,799 entries were sent to Download File (Supplementary Table S3). Three groups were classified by “AvgSpotLen”: I: <600 bp; II: 600–1600 bp; III: ≥1600 bp.

Name	Set1	Set2	Set3
AvgSpotLength	<600 bp	>=600 bp and <1600 bp	>=1600 bp
No. of runs	239,455	35,012	2332
No. of BioProjects	99	66	12
No. of runs analyzed in this study	1000	1000	1000
No. of runs with spike protein variants	627	790	932

The output files Result.csv of set1, set2 and set3 are shown in Supplementary Table S3.

References

1. Bull RA, Adikari TN, Ferguson JM, Hammond JM, Stevanovski I, Beukers AG, et al. Analytical validity of nanopore sequencing for rapid SARS-CoV-2 genome analysis. Nat Commun. 2020;11(1):6272.
2. Milne I, Stephen G, Bayer M, Cock PJA, Pritchard L, Cardle L, et al. Using Tablet for visual exploration of second-generation sequencing data. Brief Bioinform. 2013;14(2):193-202.
3. Robinson JT, Thorvaldsdottir H, Wenger AM, Zehir A, Mesirov JP. Variant Review with the Integrative Genomics Viewer. Cancer Res. 2017;77(21):e31-e4.
4. Freed NE, Vlkova M, Faisal MB, Silander OK. Rapid and inexpensive whole-genome sequencing of SARS-CoV-2 using 1200 bp tiled amplicons and Oxford Nanopore Rapid Barcoding. Biol Methods Protoc. 2020;5(1):bpaa014.
5. Baker DJ, Aydin A, Le-Viet T, Kay GL, Rudder S, de Oliveira Martins L, et al. CoronaHiT: high-throughput sequencing of SARS-CoV-2 genomes. Genome Med. 2021;13(1):21.
6. Tegally H, Wilkinson E, Giovanetti M, Iranzadeh A, Fonseca V, Giandhari J, et al. Detection of a SARS-CoV-2 variant of concern in South Africa. Nature. 2021;592(7854):438-43.
