# Supplementary material for: High-Integrity Sequencing of Spike Gene for SARS-CoV-2 Variant Determination
Source: Int J Mol Sci. 2022 Mar 17;23(6):3257. doi: 10.3390/ijms23063257 (PMC8954144; doi:10.3390/ijms23063257)
Supplement: Supplementary file 1 [file ijms-23-03257-s001.zip › Supplementary_Figures.pdf]

(A)

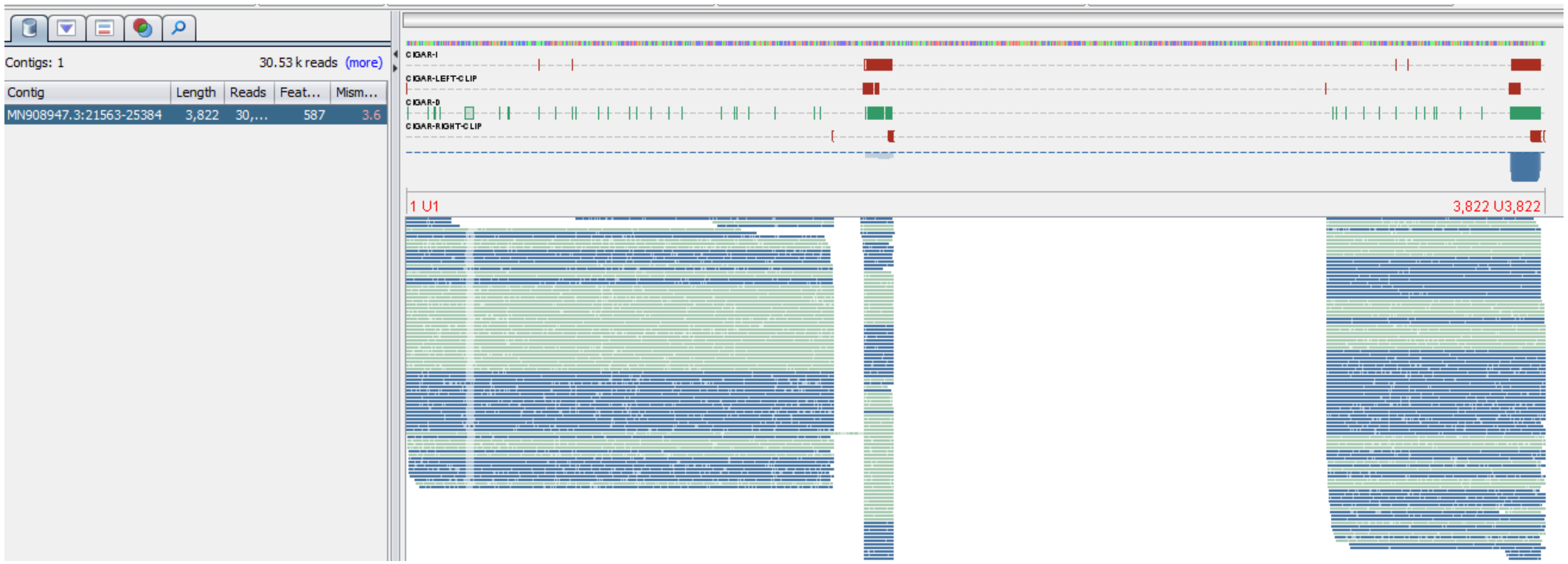

(B)

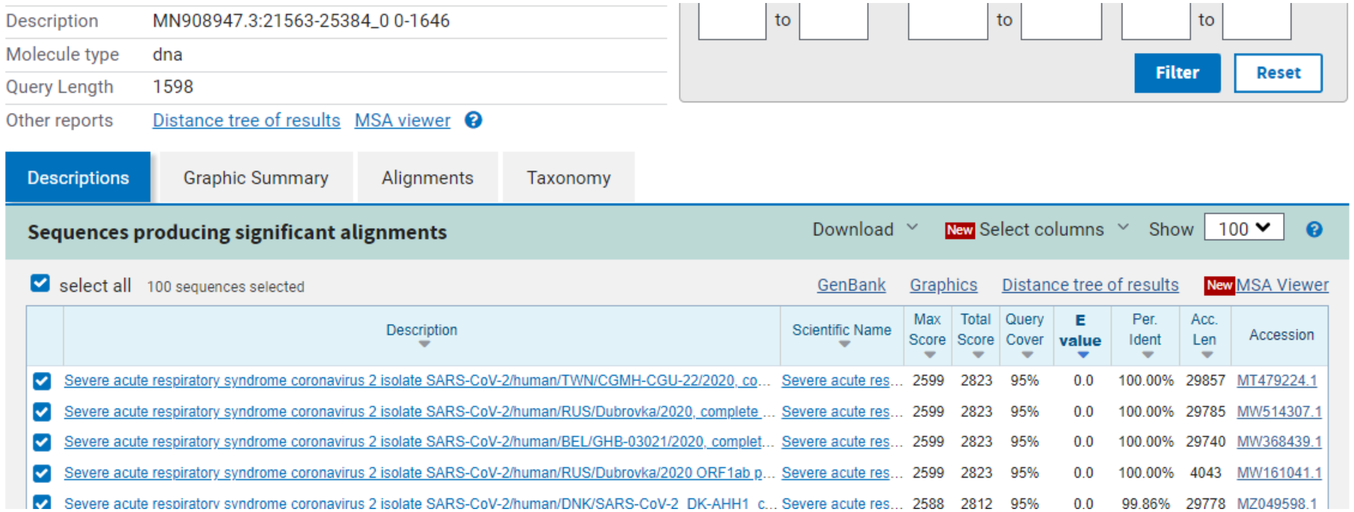

Supplementary Figure S1 (A) Read alignment of a diluted viral solution (Ct=31.79). (B) BLAST result of a consensus sequence.

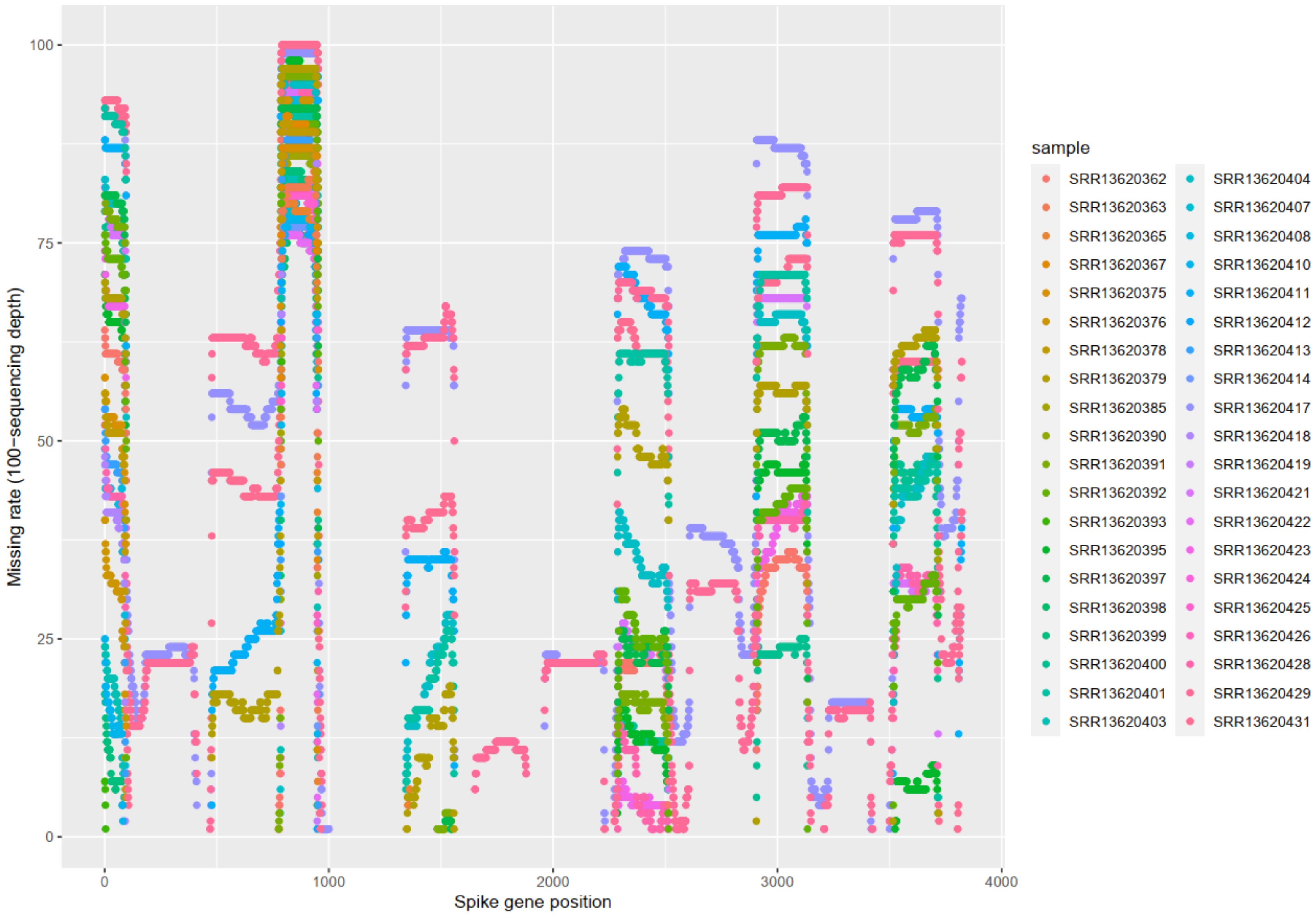

Supplementary Figure S2 Read depth distribution across spike gene. In order to highlight low sequencing depth, missing rate is defined by 100-SD and the SD is set as 100 when sequencing depth ≥100, otherwise SD=sequencing depth.
